# Molecular Dynamics model of peptide-protein conjugation: case study of covalent complex between Sos1 peptide and N-terminal SH3 domain from Grb2

**DOI:** 10.1038/s41598-019-56078-7

**Published:** 2019-12-27

**Authors:** Dmitrii A. Luzik, Olga N. Rogacheva, Sergei A. Izmailov, Maria I. Indeykina, Alexei S. Kononikhin, Nikolai R. Skrynnikov

**Affiliations:** 1 Laboratory of Biomolecular NMR, St. Petersburg State University, St. Petersburg, 199034 Russia; 2 Department of General Pathology, Institute of Experimental Medicine, St. Petersburg, 197376 Russia; 3 N.M. Emanuel Institute of Biochemical Physics, Russian Academy of Sciences, Moscow, 119991 Russia; 4 Laboratory of mass spectrometry, CDISE, Skolkovo Institute of Science and Technology, 121205 Moscow, Russia; 5 Department of Chemistry, Purdue University, West Lafayette, IN 47907 USA

**Keywords:** Peptides, Proteins, Structural biology, Molecular modelling, NMR spectroscopy, Molecular biophysics

## Abstract

We have investigated covalent conjugation of VPPPVPPRRRX′ peptide (where X′ denotes N^ε^-chloroacetyl lysine) to N-terminal SH3 domain from adapter protein Grb2. Our experimental results confirmed that the peptide first binds to the SH3 domain noncovalently before establishing a covalent linkage through reaction of X′ with the target cysteine residue C32. We have also confirmed that this reaction involves a thiolate-anion form of C32 and follows the S_N_2 mechanism. For this system, we have developed a new MD-based protocol to model the formation of covalent conjugate. The simulation starts with the known coordinates of the noncovalent complex. When two reactive groups come into contact during the course of the simulation, the reaction is initiated. The reaction is modeled via gradual interpolation between the two sets of force field parameters that are representative of the noncovalent and covalent complexes. The simulation proceeds smoothly, with no appreciable perturbations to temperature, pressure or volume, and results in a high-quality MD model of the covalent complex. The validity of this model is confirmed using the experimental chemical shift data. The new MD-based approach offers a valuable tool to explore the mechanics of protein-peptide conjugation and build accurate models of covalent complexes.

## Introduction

Various natural and modified peptides are broadly used in modern clinical practice. For example, gramicidin (topical antibiotic), insulin (life-saving diabetes treatment), oxytocin (used to induce and support labor), goserelin and degarelix (prostate cancer drugs) all fall in this category^1^. The advantages of peptide therapy are well recognized. Peptide drugs are highly selective, which minimizes their off-target interactions and lowers their toxicity. They are also affordable compared to some other classes of drugs, such as antibodies. At the same time, there is a number of limiting factors. As a rule, peptide therapeutics are not orally bioavailable. They can only be aimed at extracellular targets (the problem of effective peptide delivery into cancer cells or other target cells remains unresolved). They are also rapidly cleared from the blood through kidneys and liver. In this situation, successful peptide therapeutics are mainly analogues of natural hormones or slightly modified versions thereof^2^.

One interesting direction in this area is development of peptides that are capable of covalently binding to their targets (and thus belong to a broad class of covalent drugs). Ideally, this can be accomplished via structure-based design. Using crystallographic or NMR coordinates of (noncovalent) peptide-protein complex, one can determine the site where a covalent bond can be engineered. Subsequently, the peptide needs to be re-designed such as to incorporate a reactive group in the selected position. This group should be capable of forming a bond with one of the protein moieties that are located nearby in the structure of the complex.

The key to successful design is a proper choice of reactive group^3^. If its reactivity is too high, the peptide will bond with many randomly encountered proteins, i.e. will be wasted through off-target binding. Conversely, if the reactivity is too low, the peptide will be cleared before it has a chance to react with its target, i.e. it will not serve its intended purpose as a covalent ligand.

For a properly chosen reactive group, one expects the following scenario. First, the peptide forms a noncovalent complex with its protein target. This brings the peptide’s reactive group in close proximity to its target moiety on the protein surface, thus achieving high “local concentration of the reactants”. Under these favorable conditions, the reaction runs its course, linking the peptide to the protein.

What can be accomplished by implementing a covalent peptidic ligand for the biomedically relevant target protein? Clearly, this improves the binding affinity (formally speaking, the dissociation constant K_d_ of the covalent ligand approaches zero). It should also alleviate the situation with rapid peptide clearance. In practice, however, one may expect only a limited effect. For instance, consider a covalent peptide targeted at a certain transmembrane receptor. Prior to covalent bonding, the peptide spends much of its lifetime as noncovalent ligand (as indicated above, the conjugation chemistry must be of necessity slow). After bonding, its remaining lifespan is limited by receptor internalization and degradation. Therefore, potential gains from the use of reactive peptides are likely less than can be (naively) expected.

Nevertheless, we believe that reactive peptides can elicit a significantly different response compared to their conventional analogues. This includes changes in binding efficiency, changes in receptor-mediated endocytosis and even structural changes in the receptor triggered by covalent bonding. Along these lines, it is entirely possible that hypothetical reactive versions of goserelin or degarelix will have more favorable properties than their prototypes. Broadly speaking, development of covalent peptides adds a certain new dimension to the field of peptide therapeutics.

Recently there has been a significant amount of activity in this direction. In many cases, peptides were designed to react with cysteine thiols. The repertoire of reactive groups used in these studies included acrylamide^4–6^, chloroacetamide^7,8^ and fluoromethyl ketone^9^. However, free surface cysteines are relatively rare. Even more importantly, they are only encountered in intracellular proteins. Given the current difficulties with efficient peptide delivery into specific type cells^10,11^, this strategy may not be readily translatable into clinical practice. The attractive alternative is to employ conjugation chemistry aimed at lysine amine group. Using this line of approach, the peptides have been equipped with sulfonyl fluoride groups^12–14^ and various other types of reactive groups^12,15^. This strategy, however, also has its shortcomings – for example, sulfonyl fluoride forms unstable adducts with histidine and cysteine.

All of the studies cited above make use of intracellular protein targets (in this sense, they provide a proof of principle rather than a practical pharmacological solution). Separately, one should mention the work of Marquez *et al*.^16^ who used a peptide with dinitrofluorobenzene reactive group targeted at vascular endothelial growth factor (VEGF). VEGF circulates in blood and is a *bona fide* drug target. Furthermore, Assefa *et al*. designed a covalent version of peptidic antagonist for gonadotropin-releasing hormone receptor (GNRHR), the same receptor as targeted by goserelin and degarelix^17^. Their study, however, makes use of photoreactive azidobenzoyl group that requires ultraviolet irradiation, which is highly harmful for cells. In future, we anticipate further efforts to design covalent peptides targeted at extracellular domains of various transmembrane receptors. It can be argued that such peptides have the best chance to be successfully developed into therapeutics.

In this study, we seek to develop new modeling tools to assist in design of covalent peptide ligands. As a model system, we have chosen the complex between N-terminal SH3 domain of adaptor protein Grb2 and the peptide corresponding to the Grb2-binding sequence in the Ras guanine nucleotide exchange factor Sos1. Recently, this system has been used by Yu *et al*. to demonstrate covalent peptide binding via chloroacetamide group^8^. For the purpose of our study, we have chosen 11-residue peptide VPPPVPPRRRX′, where X′ denotes N^ε^-chloroacetyl lysine (in what follows this peptide is abbreviated Sos1-X′). We have started with a comprehensive experimental characterization of the Grb2 N-SH3 interaction with Sos1-X′. Heteronuclear NMR spectroscopy was used to confirm the noncovalent binding of this modified peptide and determine the corresponding binding constant. NMR experiments were further used to monitor the process of covalent bonding and find half-life of the reaction. As a next step, tandem mass spectrometry with collision induced dissociation was employed to confirm selective bonding of Sos1-X′ with C32 residue in Grb2 N-SH3, resulting in formation of thioether linkage. Finally, the pH dependence of the conjugation rate has been assessed by means of gel electrophoresis, confirming that the reaction proceeds through the ionic form of the thiol group.

With this information in mind, we set out to develop an MD model of Sos1-X′/Grb2 N-SH3 conjugation. As a starting point, we used the structure of noncovalent complex between Grb2 N-SH3 and Sos1 peptide^18^. In this structure, the X′ residue (N^ε^-chloroacetyl lysine) was added to the C-terminus of the peptide. The force-field parameters for X′ have been calculated using the appropriate computational tools, including quantum-chemical calculations. The obtained system is then used to conduct an MD simulation.

During this simulation we continuously monitor the distance between the chloroacetyl group in the Sos1-X′ peptide and the thiolate-anion group of residue C32 in Grb2 N-SH3. When the two groups approach each other to within a certain distance, the algorithm “flips a coin” in order to decide whether to initiate the chemical reaction.

The reaction is modeled by interpolating from one set of force field parameters to another. Initially, the simulation uses a set of force field constants pertaining to X′ and C32 (thiolate anion) residues representing two separate entities. In the end, it uses a set of constants pertinent to X′ and C32 coupled via the thioether linkage. In between the initial and the final point, the algorithm smoothly interpolates between these two parameter sets. In essence, the algorithm imitates gradual dissipation of old chemical bonds and emergence of the new ones.

The above procedure is clearly empirical. However, two aspects should be emphasized here. (1) The transition is executed sufficiently slowly such as to avoid any significant perturbations to the simulated system. MD thermostat and barostat successfully cope with the incremental changes in force field constants, so that the transition can be considered adiabatic. (2) While our protocol disregards the actual characteristics of the energy barrier separating the two states, it offers a smooth path from the known initial state (noncovalent complex) to the unknown final state (covalent complex). As a point of comparison, several well-established methods such as Empirical Valence Bond (EVB)^19,20^ or Adiabatic Reactive Molecular Dynamics (ARMD)^21,22^ usually rely on the known coordinates of the initial and final states, focusing their attention on the transition state.

Using the above protocol, we have recorded 11 MD trajectories, each of which is comprised of: (*i*) 0.5 μs conventional unrestrained simulation for the noncovalent complex, (*ii*) additional variable-length period of conventional simulation when the reaction can be initiated, (*iii*) 2 ns steered adiabatic transition to the covalent complex, (*iv*) 0.5 μs conventional unrestrained simulation for the covalent complex. In this manner, we have obtained what we believe to be a high-quality MD model of the covalent Sos1-X′/Grb2 N-SH3 complex. To validate this model, we have analyzed the NMR chemical shifts in this covalent complex. The comparison of the MD-based predictions with the experimental results confirmed (within the uncertainty margin of the predictor) the validity of the reported model.

## Results

### Noncovalent binding between Sos1-X′ and Grb2 N-SH3

Grb2 is an adaptor protein that consists of a central SH2 domain, which preferentially binds to phosphotyrosine-containing motifs, and two flanking SH3 domains, which bind to proline-rich motifs. It is an important hub in growth-factor signaling, perhaps best known for its role in Ras/MAPK pathway^23,24^. Briefly, stimulation of cell by epidermal growth factor (EGF) causes dimerization of EGF receptor (EGFR), further leading to activation of cytoplasmic tyrosine kinase domains and autophosporylation of EGFR cytoplasmic tails. Grb2 binds through its SH2 domain to two of the phosphotyrosine-containing sequences within the EGFR tails, aided by a fellow adaptor protein Shc^25^. At the same time, Grb2 binds through its two SH3 domains to proline-rich sequences within the disordered tail of the guanine nucleotide exchange factor Sos1, thus recruiting Sos1 to cell membrane and facilitating its interaction with membrane-anchored small GTPase Ras^26^. This interaction induces GDP to GTP exchange in Ras, initiating downstream signaling events that have fundamental importance for cell growth and proliferation.

Binding of Sos1 to Grb2 N-SH3 occurs through short linear proline-rich motifs^27–29^. Therefore, it can be readily characterized by solving a structure of an isolated SH3 domain in complex with the corresponding peptides. Several such structures have been determined by means of solution-state NMR^18,30,31^. It was recognized early on that these structures provide a potential basis to design therapeutics aimed at Grb2 SH3 domains^32^. However, somewhat generic character of SH3 module and the lack of high-affinity binding pockets makes it an extremely difficult target. Small-molecule inhibitors were reported for Grb2 SH3 domain, but showed poor selectivity and proved cytotoxic^33,34^. Certain progress has been achieved in designing peptidic inhibitors of Grb2 SH3 interaction with Sos1^35,36^. The most advanced peptidic constructs were equipped with protein transduction domain to enable cell entry and have had some success in preclinical studies on mice^37^.

Very recently, Yu and co-workers developed a family of covalent peptidic ligands targeted at Grb2 N-SH3^8^. In doing so, they took advantage of the sole cysteine residue C32, which is favorably positioned on the surface of Grb2 N-SH3 near the peptide-binding site, and relied on chloroacetamide group to conjugate the peptide to the corresponding thiol. Taking a cue from their work, we used a slightly longer peptide sequence, VPPPVPPRRR, for which the PDB coordinates of the complex with Grb2 N-SH3 are available^18^. We further added N^ε^-chloroacetyl lysine residue (X′) in the C-terminal position, arriving at the peptide termed Sos1-X′.

First, we set out to characterize the interaction between Sos1-X′ and Grb2 N-SH3 experimentally. This interaction involves rapid formation of noncovalent complex (denoted SH3·Sos1-X′), followed by its gradual progression to covalent complex (denoted SH3:Sos1-X′). To separate the former process from the latter, we have engineered the C32S mutant of Grb2 N-SH3, which is incapable of covalent bonding with Sos1-X′. Previously it has been shown that this conservative cysteine-to-serine mutation, which involves the surface residue outside the ligand-binding interface, has no effect on structural integrity and ligand binding properties of Grb2 N-SH3^38^.

**Figure 1:**
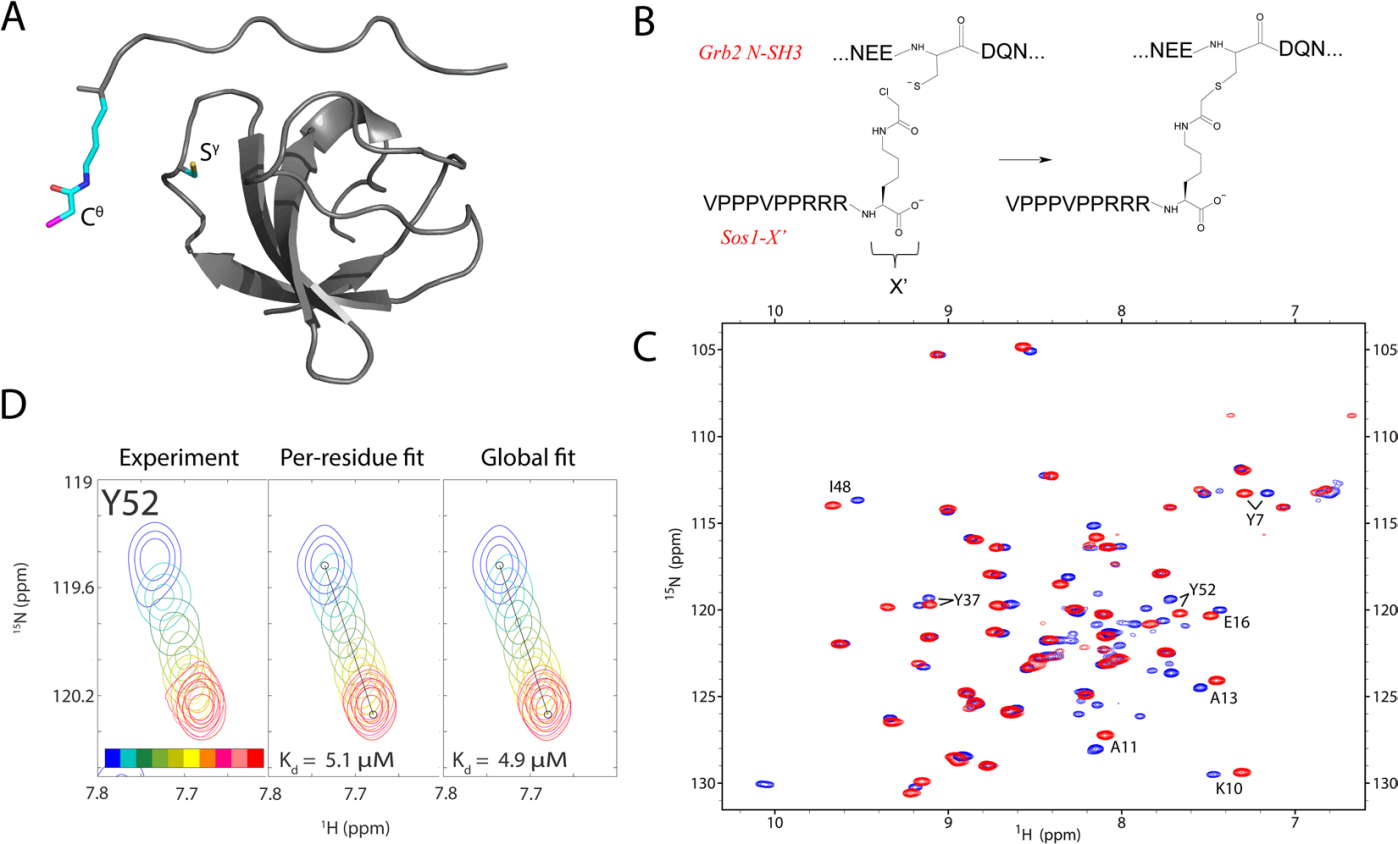
(**A**) Model of the noncovalent complex SH3·Sos1-X′ based on the PDB structure 1GBQ^18^. Side chains of residues X′ and C32 are shown in stick representation. (**B**) Composition of the peptide Sos1-X′ and its expected reaction with SH3 residue C32. (**C**) Superposition of the ^1^H^N^,^15^N-HSQC spectra of apo C32S SH3 (blue) and C32S SH3·Sos1-X′ (red). The 8 pairs of peaks that have been used to determine the *K*_*d*_ constant are labeled in the plot. (**D**) ^1^H^N^,^15^N-HSQC titration for the peak from SH3 residue Y52: experimental results (left panel), best fit by the TITAN software^39^ using Y52 data only (middle panel) or, alternatively, using the entirety of the data for 8 titrating resonances (right panel). The color-coding of the spectra and other details are described in the caption of Fig. S1.

To determine *K*_*d*_ constant of noncovalent binding between Sos1-X′ and C32S Grb2 N-SH3, we conducted ^1^H^N^,^15^N-HSQC titration experiment. Adding the peptide caused moderate shifts for a number of SH3 peaks corresponding to the residues on peptide-binding interface (see Fig. 1C)^18^. The data are consistent with fast-to-intermediate exchange between the two states of the SH3 domain (free and peptide-bound). For quantitative analysis, we have selected a group of 8 well-resolved peaks showing substantial titration effects. The two-dimensional HSQC titration data for these peaks have been fitted using the program TITAN^39^ on per-residue basis, as well as collectively (illustrated in Fig. 1D; for complete summary see Fig. S1). The collection of per-residue fits produced the average *K*_*d*_ value of 4.6 ± 2.3 μM, while the global fit yielded 4.9 μM. These two results are obviously consistent with each other. More importantly, they are similar to the dissociation constant previously determined for the unmodified Sos1 peptide, *K*_*d*_ = 3.5 μM. Indeed, the reactive residue X′ has been appended to the C-terminus of the Sos1 peptide, where it is projected into solvent and therefore should not interfere with the binding (see Fig. 1A,B). We conclude that Sos1-X′ forms a *bona fide* noncovalent complex with Grb2 N-SH3, in agreement with the predicted mechanism of covalent conjugation. Considering this result, as well as the chemical shift mapping data, it is safe to suggest that SH3·Sos1-X′ complex is identical to the SH3·Sos1. Thus, the existing structure of SH3·Sos1 provides a good starting point to build a model of the covalent complex SH3:Sos1-X′ (described in what follows).

It is also worth noting that addition of the peptide considerably improves the quality of the spectral map, leading to more uniform peak intensities. The improvement is particularly significant at higher sample concentration, 1 mM (see Fig. S2A). This suggests that apo SH3 domain suffers from weak self-association, similar to what has been described before for Crk SH3 domain^40^. It has also been shown that peptide binding leads to reduction in μs-ms dynamics, which is detectable at several sites in Src SH3 domain, and improves protection against solvent exchange^41^. Finally, it is worth mentioning that in the case of α-spectrin SH3 domain, peptide binding leads to a moderate increase in thermodynamic stability of the domain^42^.

### Covalent binding between Sos1-X′ and Grb2 N-SH3

**Figure 2:**
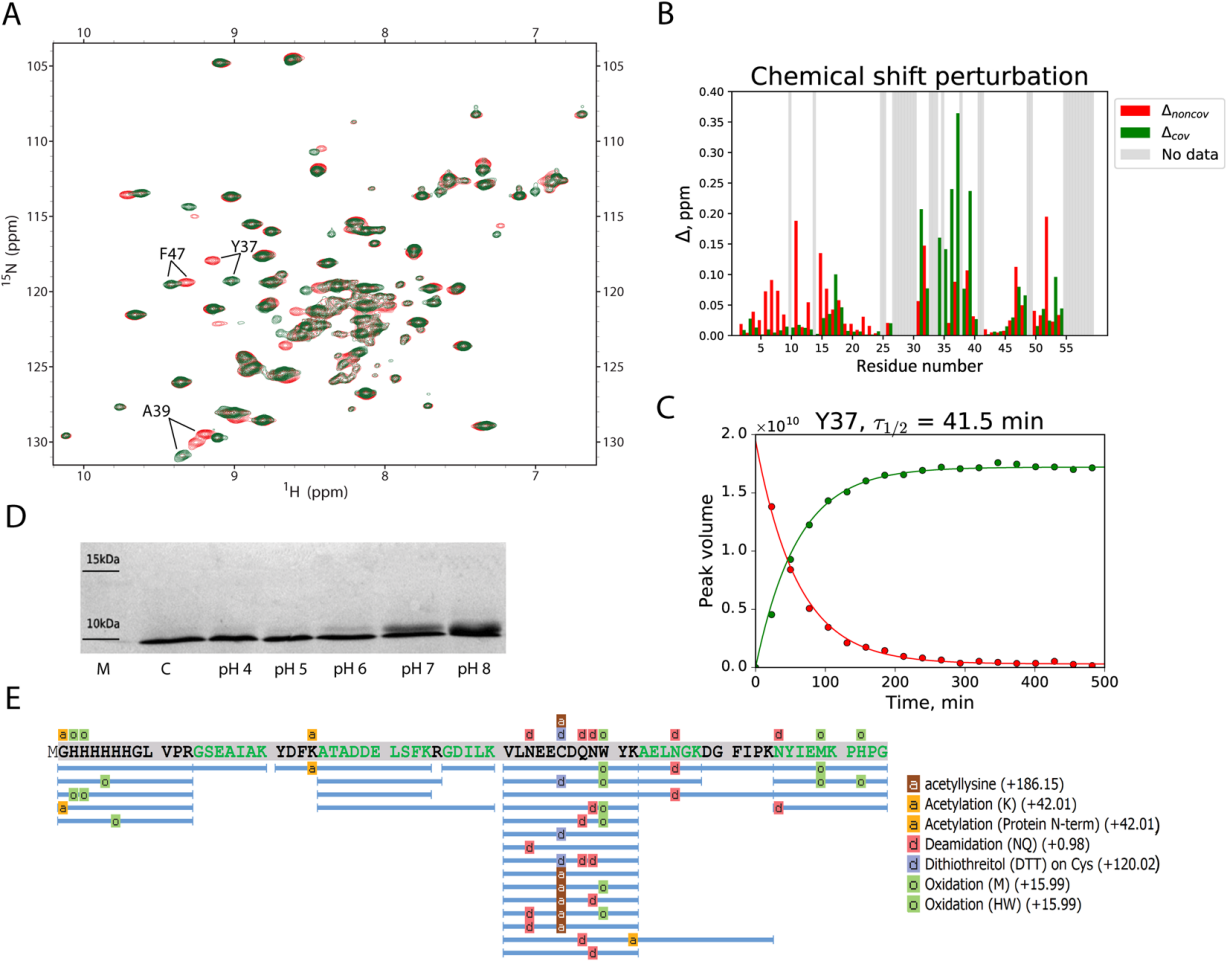
(**A**) Superposition of the two ^1^H^N^,^15^N-HSQC spectra from the sample containing 1 mM ^15^N-labeled SH3 and 2 mM unlabeled Sos-1X′. The spectra were recorded during the time interval 8–32 min after initiation of the reaction (red) and 8 hrs later (green). The 3 pairs of peaks that have been used to determine the *τ*_1/2_ constant are labeled in the plot. (**B**) Chemical shift differences between (*i*) noncovalent SH3·Sos1-X′ complex and apo SH3 (Δ_*noncov*_, red bars) and (*ii*) covalent SH3:Sos1-X′ complex and SH3·Sos1-X′ (Δ_*cov*_, green bars). Grey areas indicate the absence of data (proline residues, spectral overlaps). (**C**) Kinetic curves for residue Y37 reflecting the decrease in the intensity of the spectral peak associated with SH3·Sos1-X′ species (red points) and the concomitant increase in the intensity of the peak associated with SH3:Sos1-X′ species (green points). The time points in the graph correspond to the mid-points of the respective HSQC experiments. The best-fit *τ*_1/2_ value is indicated above the plot. (**D**) SDS-PAGE data reporting on the outcome of Sos1-X′ conjugation to SH3 domain. Reaction time 20 min, the pH conditions are marked underneath the lanes. (**E**) Sequence coverage based on LC-MS/MS experimental data and PEAKS Studio interpretation. Tryptic peptides with modifications are labelled.

To investigate the formation of covalent complex SH3:Sos1-X′, we have used the sample containing 1 mM of ^15^N-labeled wt-SH3 and 2 mM of Sos1-X′. The progression of the conjugation reaction was monitored by recording a series of back-to-back ^1^H^N^,^15^N-HSQC spectra. The first spectral map (red spectrum in Fig. 2A) contains a set of strong signals corresponding to noncovalent complex SH3·Sos1-X′. It also contains a set of weak peaks from covalent complex SH3:Sos1-X′ which has formed during the course of the NMR experiment. The last map acquired 8 h after the start of the reaction (green spectrum in Fig. 2A) contains solely SH3:Sos1-X′ signals. If any amount of unreacted SH3 domain remains in the sample, it is too small to be reliably detected in the spectrum.

The inspection of Fig. 2A immediately shows that the perturbations to the spectrum due to covalent binding are minimal. This leads us to conclude that engineering of the covalent bond in the SH3·Sos1-X′ complex leaves its structure essentially intact. This is not surprising given that X′ residue is positioned opposite to C32 so that they can form a thioether linkage without causing any significant distortions to the structure. It appears that only minimal structural adjustments are needed to accommodate the newly formed covalent bond.

To localize small structural changes caused by peptide conjugation, we analyzed chemical shift differences between SH3·Sos1-X′ and SH3:Sos1-X′ spectra. For this purpose, we have combined ^1^H^N^ and ^15^N shifts, as often done in the context of chemical shift mapping^43^:

1.1 $${\Delta }_{{noncov}}={[0.5\cdot {({\delta }_{HN}^{{noncov}}-{\delta }_{HN}^{apo})}^{2}+0.07\cdot {({\delta }_{N}^{{noncov}}-{\delta }_{N}^{apo})}^{2}]}^{1/2},$$

1.2 $${\Delta }_{cov}={[0.5\cdot {({\delta }_{HN}^{cov}-{\delta }_{HN}^{noncov})}^{2}+0.07\cdot {({\delta }_{N}^{cov}-{\delta }_{N}^{noncov})}^{2}]}^{1/2}.$$

Δ_*noncov*_ and Δ_*cov*_ were further determined under the identical sample conditions, cf. Fig. S2. The summary of the results is shown in Fig. 2B. The inspection of this graph suggests that larger values of Δ_*cov*_ are found near the attachment point, i.e. in the n-Src loop housing residue C32. This is understandable, since peptide conjugation alters the covalent bonding pattern at the attachment site and, furthermore, is likely to have some effect on the conformation (and conformational dynamics) of the n-Src loop. Smaller shifts Δ_*cov*_ are also observed in many of the same sites where Δ_*noncov*_ were detected. Indeed, we expect that covalent bonding entails certain subtle adjustments at the protein-peptide interface. This is reflected in the chemical shifts of the same residues that previously proved sensitive to (noncovalent) peptide binding. The effect is additionally illustrated in Fig. S3 that shows the mapping of Δ_*cov*_ and Δ_*noncov*_ onto the structure of the complex.

The series of back-to-back HSQC spectra, two of which are shown in Fig. 2A, can also be used to characterize the kinetics of the conjugation reaction. Given the experimental sample conditions and the *K*_*d*_ constant reported above, it follows that SH3 domain is fully loaded with the peptide (the proportion of the apo SH3 does not exceed 0.4%). Therefore, the reaction can be described simply as a gradual transformation of SH3·Sos1-X′ into SH3:Sos1-X′, obeying the first-order exponential kinetics. To quantify this process, we have selected 3 residues which give rise to well-resolved peak pairs assignable to the noncovalent and covalent complex. Fitting these data on per-residue basis leads to the average value *τ*_1/2_ = 41.6 ± 1.9 min for half-life of the reaction, while the global fitting yields 41.5 min (illustrated in Fig. 2C, summarized in Fig. S4). If viewed from the perspective of potential diagnostic or therapeutic uses, this is a favorable outcome. Indeed, as commented in the Introduction, the conjugation chemistry should not be too fast or too slow. The timeframe of ca. 1 h appears to be the desired middle ground.

While NMR data strongly support the proposed conjugation mechanism, they offer no definitive proof that the reaction occurs as intended. For such proof, we turn to liquid chromatography/tandem mass spectrometry. The sample of SH3:Sos1-X′ for LC-MS/MS analysis was prepared in a similar manner to the NMR study, then transferred to ammonium bicarbonate buffer and subjected to trypsinolysis. The trypsin treatment is expected to cleave between residue R10 in the peptide and the X′ derivate that is conjugated to the protein (see Fig. 1B). Consequently, in the tryptic digest C32 residue should carry a modification with the mass 186.15 (acetyllysine). Detection of this particular modification should prove that the reaction has occurred as expected.

The fragmentation spectra of SH3:Sos1-X′ produced a reasonably good coverage of the SH3 sequence, see Fig. 2E. The expected acetyllysine modification is consistently found in the position C32, thus confirming the presence of the intended thioether linkage. Note that in some of the tryptic peptides C32 remains unmodified, while in others it carries a dithiothreitol modification (the consequence of 2 mM DTT in the reaction medium). Bear in mind, however, that these data should not be interpreted as a measure of reaction efficiency since LC-MS/MS method is not suitable for product quantification. In fact, NMR data indicate that the efficiency of the conjugation reaction approaches 100% (see above). Similarly, the presence of oxidized Met, His and Trp residues, deamidated Gln and Asn residues, etc. in the tryptic digest does not reflect the true state of the sample, but largely stems from the experimental artefacts^44^.

We have also conducted an experiment to shed light on the mechanism of the conjugation reaction. Specifically, we carried out the reaction under different pH conditions, employing SDS-PAGE to detect the adduct (see Fig. 2D). The observed increases in the reaction rate with increasing pH confirm that the reaction proceeds via thiolate-anion form of C32, in agreement with the well-established mechanism of a small-molecule reaction involving chloroacetamide and cysteine^45^. This observation proved very important for our subsequent modelling efforts (see below). Generally, chloroacetamide chemistry is known for its high degree of specificity toward cysteine residue^46^. Indeed, our mass spectra showed no evidence of off-target modifications, involving e.g. proximal lysine sites. Our NMR spectra, featuring one set of intense spectral peaks assignable to the SH3 domain, also confirm that the covalent complex is formed uniquely through the target C32 residue.

Finally, we were interested in folding properties of SH3:Sos1-X′ vs. SH3·Sos1-X′. It is a common knowledge that small domains, such as Grb2 N-SH3, have an ability to fold spontaneously. On a fundamental level, this property is a product of evolutionary pressure – simple structural motifs have evolved to fold independently (without help from chaperonins). On the other hand, this argument does not apply to the covalent complex of SH3:Sos1-X′. Indeed, its polypeptide chain, featuring the thioether isopeptide linkage, is a wholly artificial construct. Therefore, it is not *a priori* clear whether SH3:Sos1-X′ retains the ability to spontaneously fold similar to the apo SH3 domain.

In order to answer this question, we have characterized the ability of SH3:Sos1-X′ to refold following thermal denaturation. The sample of noncovalent SH3·Sos1-C complex was used as a reference. Our results demonstrate that both covalent and noncovalent complexes can successfully refold after 30 minutes at 70 °C with only moderate losses (see Fig. S5). Similarly, the covalent complex refolds after 30 minutes at 90 °C, which is well above its melting temperature^47^. While non-native covalent linkages can affect protein folding, especially in homodimers^48^, this is apparently not the case in this particular peptide-protein conjugate.

### MD simulation of covalent binding between Sos1-X′ and Grb2 N-SH3

In this section, our objective is to start with the known structure of the noncovalent SH3·Sos1-X′ complex and by means of the specially designed MD algorithm transform it into covalent complex SH3:Sos1-X′. In doing so, we seek to obtain a maximally faithful model of SH3:Sos1-X′.

As a first step, we calculated Amber ff14SB force-field^49^ parameters for non-native residues in this system: N^ε^-chloroacetyl lysine (X′) and its adduct with cysteine. For this purpose, a standard procedure has been employed (see Materials & Methods). In brief, small fragments have been chosen to represent the residues of interest and geometry-optimized by means of DFT calculations. The obtained geometries were used to calculate electrostatic potentials by means of Hartree-Fock method; these potentials were in turn used to fit partial charges via the RESP scheme^50^. Other lacking force-field parameters have been taken from GAFF2 force field^51^. In this manner, we have parameterized N^ε^-chloroacetyl lysine (termed LYC), C-terminal N^ε^-chloroacetyl lysine (CLYC), adduct of N^ε^-chloroacetyl lysine with cysteine (LYZ), adduct of C-terminal N^ε^-chloroacetyl lysine with cysteine (CLYZ) and adduct of cysteine with N^ε^-chloroacetyl lysine (CYZ). The boundary between the covalently bonded CYZ and LYZ (or CLYZ) residues is across the S^γ^-C^θ^ bond (see Fig. 1B). Given the C-terminal position of N^ε^-chloroacetyl lysine in the Sos1-X′ peptide, only CLYC, CLYZ and CYZ residues are required for further simulations.

To build the initial model, we begin with the structure PDB ID 1GBQ representing noncovalent complex between Sos1 and Grb2 N-SH3^18^. 1GBQ is a minimized average NMR structure and, as such, is expected to be less accurate than crystallographic structures^52^. Nevertheless, comparing it with x-ray coordinates of apo Grb2 N-SH3 PDB ID 6SDF indicates a very good level of agreement (*rmsd* 0.57 Å for the backbone atoms, 0.72 Å overall). Beginning with 1GBQ, we appended X′ (CLYC) residue to the extended C-terminus of the Sos1 peptide using the standard facilities of Amber 16^53^. Finally, we assigned the (standard) type CYM to residue C32 in the SH3 domain to simulate the ionized form of cysteine. As discussed in the previous section, conjugation of N^ε^-chloroacetyl lysine to cysteine occurs through the thiolate-anion form of cysteine.

The resulting structure has been placed in TIP3P water box, energy-minimized and heated to the temperature 293 K. This configuration was then used to launch 11 MD trajectories (starting with different initial velocity distributions). Over the first 500 ns, each trajectory was recorded as a regular, conventional MD simulation. Beginning from this point, the system is deemed reactive: i.e. it is assumed that X′ and C32 may form a bond if the two reactive groups come sufficiently close to each other. Accordingly, the algorithm starts to monitor the distance between C^θ^ (X′) and S^γ^ (C32) atoms. This distance varies significantly during the course of the simulation, reflecting conformational mobility of (*i*) flexible arginine-rich peptide tail and (*ii*) long N^ε^-chloroacetyl lysine side chain. When this distance falls below *r*_*c*_ = 3.3 Å, it is assumed that the two reactive groups are within the capture radius of each other and, therefore, the conditions exist for the chemical reaction to occur.

This does not mean, however, that the reaction is triggered automatically upon crossing the *r*_*c*_ threshold. Instead, the algorithm makes a decision on whether to initiate the reaction by means of rejection sampling (i.e. essentially by flipping a coin, see Materials & Methods). The goal of this scheme is to lower the reaction rate. While it is impossible to reproduce the correct time scale of the reaction, ca. 1 h, this approach is meant to ensure that the simulated reaction is slow on the scale of conformational dynamics in SH3·Sos1-X′.

**Figure 3:**
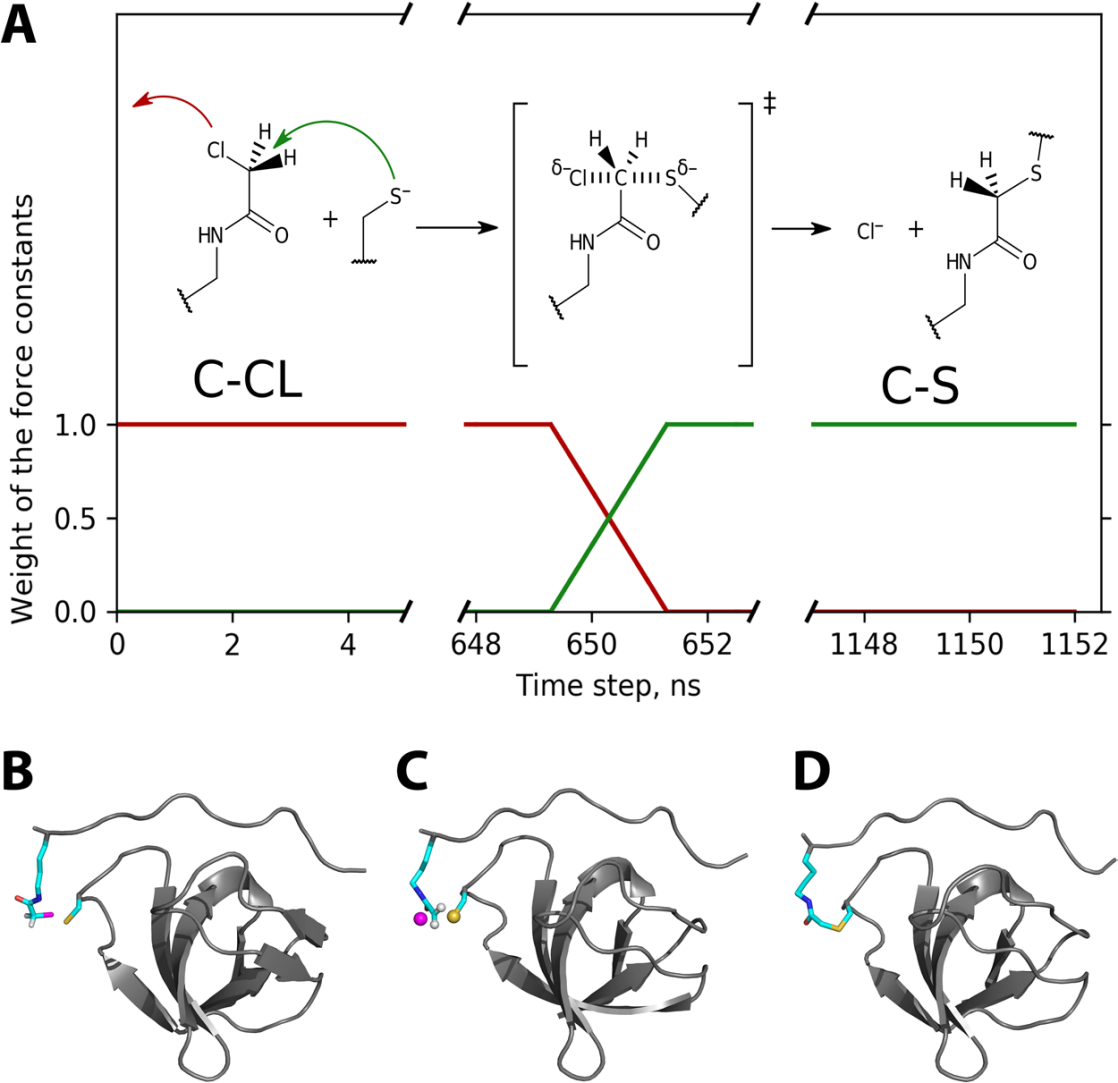
(**A**) Schematic illustration of the algorithm used to model Grb2 N-SH3 conjugation to Sos1-X′. Scaling factors applied to the force constants for C^θ^-Cl and C^θ^-S^γ^ bonds are shown as red and green profiles, respectively. The timeline corresponds to trajectory #4 out of eleven. The detailed flowchart of the MD algorithm is shown in Fig. S6. (**B**–**D**) MD frames corresponding to the beginning, the midpoint and the end of the T_*rxn*_ period in trajectory #4. The snapshot in the middle shows four atoms in a transition-state arrangement, cf. the reaction scheme in the top portion of panel (A). Interestingly, our simple MD method is likely to (approximately) capture the orientational dependence of the reaction. Indeed, it predicts that S^γ^ atom should approach C^θ^ from the side opposite of Cl (both sulfur and chlorine atoms are bulky and carry partial negative charges).

At the moment when the reaction is initiated, a special algorithm takes control. This algorithm makes use of two topologies and their associated sets of force-field parameters: one pertaining to the noncovalent complex SH3·Sos1-X′ (involving residues CLYC and CYM), and the other pertaining to the covalent complex SH3:Sos1-X′ (involving covalently bonded CLYZ and CYZ). The algorithm interpolates between these two parameter sets. Specifically, the force constants from the first set are gradually reduced to zero, while the force constants from the second set are gradually ramped up to their full value. As an example, we illustrate the scaling of the force constants associated with C^θ^-Cl bond in the chloroacetyl group and C^θ^-S^γ^ bond in the thioether bridge (red and green profiles in Fig. 3A, respectively). It can be said that the first bond is gradually dissolved, while the second is gradually materialized.

The transition between the initial SH3·Sos1-X′ state and the final SH3:Sos1-X′ state is conducted over the time interval T_*rxn*_ = 2 ns. During this period, the linear interpolation between the two states is accomplished through (*i*) consecutive regeneration of topology files and (*ii*) using scalable artificial restraints that emulate certain force field terms (see Materials & Methods for details). By the end of the transition period, the artificial restraints are replaced with the equivalent force field terms for SH3:Sos1-X′.

After the formation of SH3:Sos1-X′ is completed, it is simulated in a conventional fashion under the control of the regular force field. In this final stage of our protocol, we record 500 ns of such conventional MD trajectory.

In what follows, we discuss the behavior of the 11 MD models that have been generated in this manner. The initial 500-ns-long portion of each trajectory corresponds to the conventional MD simulation of the SH3·Sos1-X′ complex. The dynamics of the complex is in line with expectations. The polyproline-II segment of Sos1 (VPPPVPP) remains wedged into two shallow hydrophobic grooves on the surface of the SH3 domain, maintaining key hydrogen bonds to the side chains of the conserved W36 and Y52 residues (these and other essential residues are visualized in Fig. S7)^40,54^. The first arginine following the PPII element, R8, also maintains a strong connection to the SH3 domain through two concurrent salt bridges – to D15 and E16. The following two arginines, R9 and R10, are highly dynamic, with side chains mostly projected into solvent. However, occasionally these arginines also form salt bridges with different anionic residues in the RT and n-Src loops of the SH3 domain; these salt bridges often turn out to be rather long-lived (100 ns or longer). Finally, the long side chain of N^ε^-chloroacetyl lysine (X′) shows a similarly diverse behavior – most of the time it is extended into solvent, but occasionally it inserts itself between the RT and n-Src loops and remains in this configuration for extended periods of time (100 ns or longer). Accordingly, the distance between C^θ^ atom in X′ chloroacetyl group and S^γ^ atom in C32 thiolate group shows a wide range of variation during the course of the simulations (between 3.1 and 32.8 Å).

After the time point 500 ns, the simulation of SH3·Sos1-X′ is continued in the same conventional fashion up to the moment when the reaction is initiated. This is illustrated for one of the trajectories in Fig. 3A, where the reaction is started at time point 649.3 ns. Considering all trajectories, the conjugation occurs in the time window between 513 and 1,047 ns.

We will now discuss in more detail the events that happen during the course of the “reaction”, i.e. during the time T_*rxn*_ = 2 ns. It begins with a configuration where C^θ^ atom is maximally close to S^γ^, see Fig. 3B. Initially, the system evolves similar to a noncovalent complex, with only minimal extraneous restraints. However, after 1 ns a “transition state” is formed, where the force field terms representing separate X′ and C32 residues are balanced with the other force field terms representing the thioether linker. This configuration, illustrated in Fig. 3C, involves pentavalent carbon C^θ^ and appears curiously realistic. Indeed, our algorithm, which involves gradual dissolution of old bonds and simultaneous emergence of new bonds, is expected to produce a reasonably looking “transition state”. Of course, the similarity to the actual transition state is rather superficial (building a realistic model of transition state would require quantum-chemical calculations, which are not a part of our procedure).

From the “transition state” the system evolves toward the *bona fide* thioether bond, see Fig. 3D. The chlorine atom leaves and diffuses into solvent (in a form of chloride anion). At the end of the T_*rxn*_ period the force field terms corresponding to the old covalent geometry are reduced to nil and the terms corresponding to the new covalent geometry are brought to their full intensity. Beginning from this point the simulation of the newly formed SH3:Sos1-X′ complex is continued in a conventional fashion. The length of this final portion of the trajectory is 500 ns (same as the initial portion of the trajectory dedicated to SH3·Sos1-X′). The entire sequence of events is visualized in Video S1.

At this point it is appropriate to discuss the setting of the two parameters used in our computational scheme, *r*_*c*_ = 3.3 Å and T_*rxn*_ = 2 ns. Both values represent a heuristic compromise. In the case of *r*_*c*_, the value smaller than 3 Å would mean that prohibitively long simulations are required to achieve the bonding. Conversely, large values that are greater than ca. 5–10 Å would mean that the system is actively steered to form a bond, even when its current conformation is not conducive to bond formation. In the case of reaction time, a short T_*rxn*_ period would result in an explosive growth of potential energy and termination of the trajectory. (The limiting case T_*rxn*_ = 0 corresponds to a scenario when one imposes a full-strength bond on a pair of distant atoms, which causes the simulation to immediately “blow up”). On the other hand, using long T_*rxn*_ would mean that the system has a long time to evolve while in a somewhat artificial state, such as the “transition state” described above. This is also undesirable.

**Figure 4:**
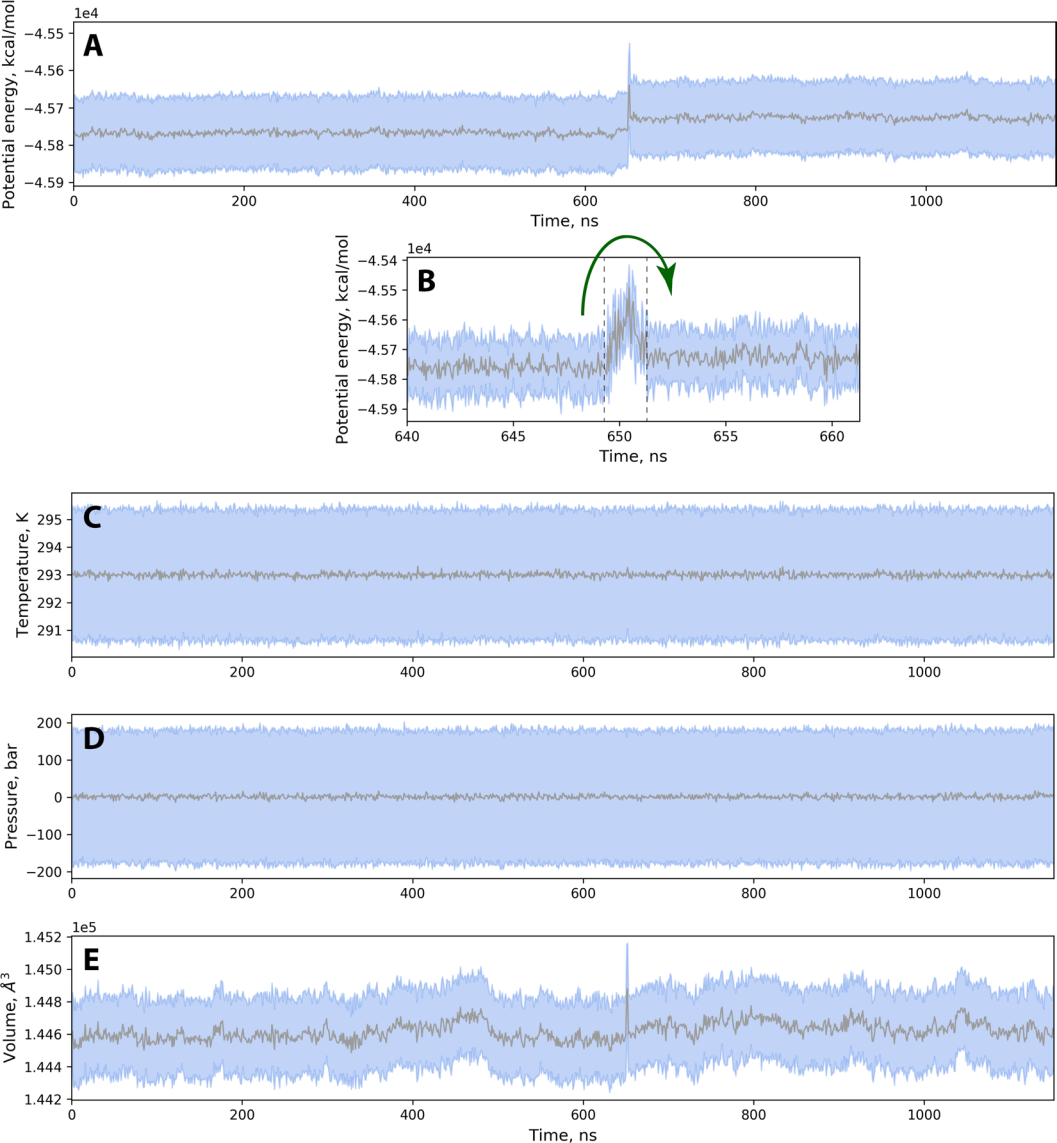
(**A**) Time trace of potential energy for trajectory #4. Each point in the graph represents an average over 1 ns interval; the *rmsd* calculated over the same time interval is shown in a form of a blue band. (**B**) Expansion of the potential-energy plot focusing on the transition region (the beginning and the end of the T_*rxn*_ period are marked by the vertical dashed lines); the sampling interval of 50 ps is used in this panel. (**C**–**E**) Time traces of temperature, pressure and volume for trajectory #4.

The force field terms that are ramped up/down in our protocol involve a small part of the simulated system (specifically, just a few atoms). Furthermore, these terms are ramped up/down sufficiently slowly over the time interval 2 ns. Consequently, the potential energy of the system increases only modestly during the transition. This is illustrated in Fig. 4A – the transient increase in potential energy corresponds to only 0.5% of the total. This is of similar magnitude to random fluctuations of potential energy (represented in the graph by a pale blue band). From this perspective the transformation can be characterized as almost adiabatic (i.e. no heat is exchanged with the environment)^55^.

On a more quantitative level, changes in potential energy should, of course, be reflected in kinetic energy of the system. However, the situation is successfully handled by the thermostat so that the temperature remains stable throughout the transition period, see Fig. 4C (i.e. the thermostat removes the excess heat). Similarly, the barostat successfully maintains constant pressure, Fig. 4D. The only macroscopic parameter that becomes noticeably perturbed in this NPT simulation is the volume of the simulation cell, Fig. 4E. However, the observed transient increase in volume does not exceed 0.15% of the total. This is similar to volume fluctuations during the course of the regular trajectory. The observed spike is short-lived – the volume returns to its original value by the end of the T_*rxn*_ period. Therefore, we conclude that our scheme avoids any appreciable perturbations to the essential macroscopic parameters: temperature, pressure and volume. This outcome validates our choice of T_*rxn*_ and, more broadly, the entire scheme used to model Grb2 N-SH3 conjugation with Sos1-X′.

### Evaluation and validation of the model

Each of our 11 simulations described in the previous section includes the 500-ns final segment that represents the covalent complex SH3:Sos1-X′. Immediately following the bond formation, the system may need some time to relax and dissipate the residual structural strain. Therefore, we choose to ignore the first 50 nanoseconds subsequent to T_*rxn*_ and focus on the final 450-ns interval. Thus, we have 11 × 450 ns = 4.95 μs of conventional MD simulations that can be viewed as an MD model of the SH3:Sos1-X′ complex.

Similarly, the initial part of each simulation is representative of the noncovalent SH3·Sos1-X′ complex. Ignoring the first 50 ns, the net duration of the relevant MD “footage” is 4.95 μs + 2.27 μs (the latter contribution is from the period prior to bond formation, when we monitor the C^θ^-S^γ^ distance, but aside from that record the trajectory in a conventional manner). Therefore, we have a total of 7.22 μs of conventional MD simulations that can be regarded as an MD model of the SH3·Sos1-X′ complex. This presents an opportunity to compare the two MD models, SH3·Sos1-X′ and SH3:Sos1-X′, and thereby assess the effect of conjugation on protein structure.

**Figure 5:**
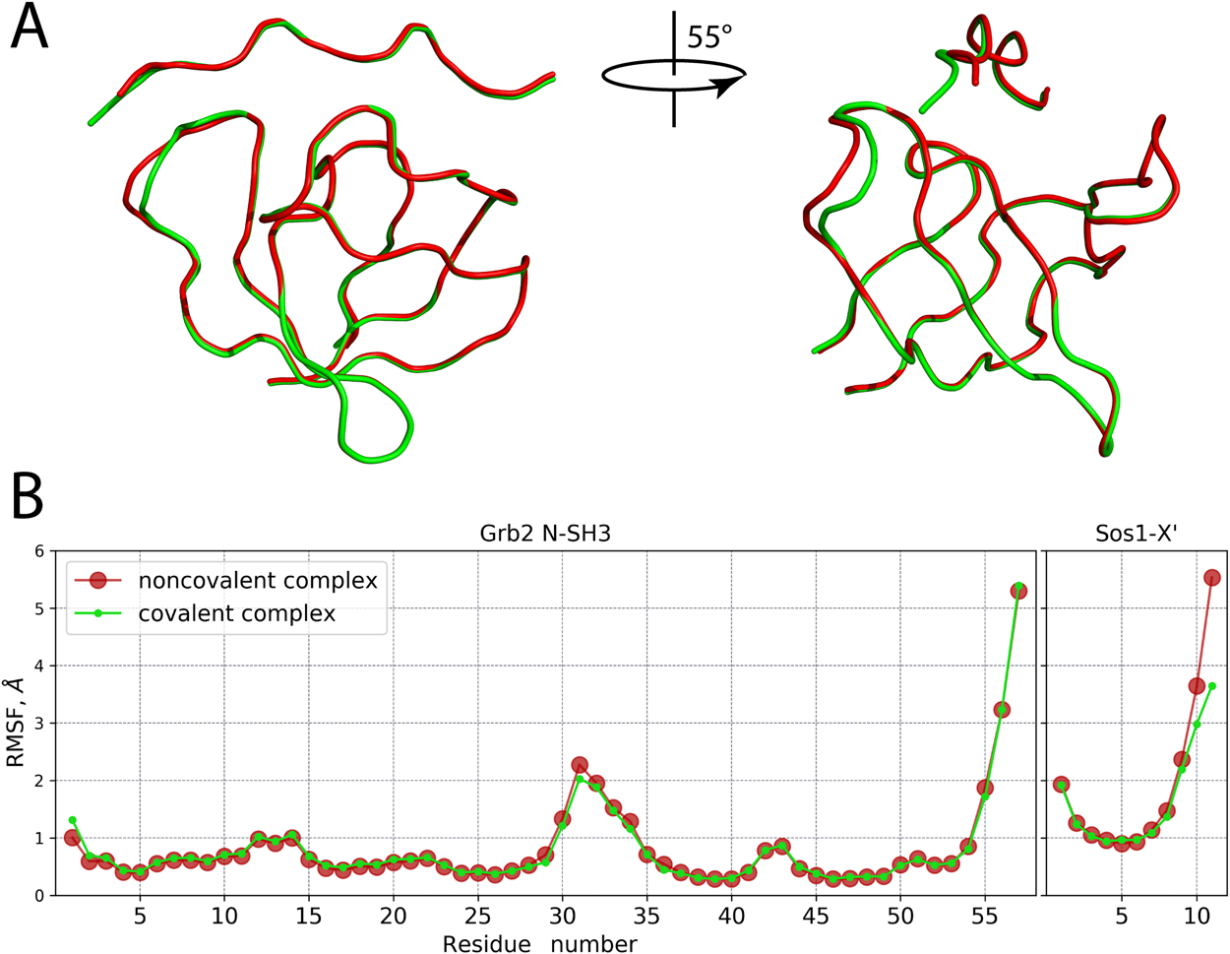
(**A**) Superposition of C^α^ traces representing the average coordinates of the MD models of SH3·Sos1-X′ and SH3:Sos1-X′ (red and green color, respectively). The perspective in the right portion of the plot offers a clear view of conformational changes in the n-Src loop. For positions of individual residues, consult Fig. S7. (**B**) Root mean square fluctuation (*rmsf*) characterizing the mobility of C^α^ atoms in the MD models of SH3·Sos1-X′ and SH3:Sos1-X′. Prior to calculating *rmsf* all MD snapshots were superimposed with respect to C^α^ atoms from the secondary-structure regions of the SH3 domain.

Figure 5A shows the superposition of the backbone traces from the average MD coordinates of SH3·Sos1-X′ and SH3:Sos1-X′ complexes (painted red and green, respectively). Clearly, there is very little difference between the two models. In fact, the differences are localized in the n-Src loop region of the SH3 domain and the C-terminus of the Sos1-X′ peptide – i.e. precisely at the conjugation site. In particular, the C^α^ atom of residue C32 is displaced by 1.7 Å, while C^α^ atom of residue X′ is displaced by 3.1 Å. We conclude that the structure adapts to covalent bonding through local conformational changes involving only a few residues proximal to the conjugation site.

Not surprisingly, the key peptide-protein interactions also remain intact, e.g. hydrogen bond between Y52 and P3 (present in 97% of all MD frames in both SH3·Sos1-X′ and SH3:Sos1-X′ models) as well as several other conserved hydrogen bonds. Similarly, the “fuzzy” electrostatic interactions between the arginine-rich tail of Sos-1X′ peptide and the negatively charged SH3 patches^40^ are maintained as well. However, the pattern of these transient contacts is somewhat changed. In particular, the salt bridges between R8 and D15, E16 are slightly weakened as a result of covalent linking. R9 tends to interact less with D15, E31, C32 and more with D33, Q34, whereas R10 interacts less with E16 and more with D15. Finally, X′ residue shows some hydrogen bonding to carbonyl oxygen of C32, with which it is linked via thioether bridge. The hydrogen bonds are through backbone N atom (24%), as well as N^ζ^ atom in the linker (12%).

In addition, we have also analyzed the amplitudes of backbone motion in the MD models of SH3·Sos1-X′ and SH3:Sos1-X′. The results are shown Fig. 5B. The observed differences are minor and limited to the C-terminal tail of the peptide, plus essentially a single residue in the n-Src loop, viz. E31. Interestingly, the loop remains mobile despite covalent linking – apparently, long and flexible linkage allows it to retain a significant level of motional freedom. Thus, both structure and dynamics of the complex are only minimally affected by the covalent linking.

We have also sought to validate our SH3:Sos1-X′ model using experimental data. The optimal strategy would be to compare our model to a crystallographic structure of the covalent complex. We attempted to obtain such structure in collaboration with S. Korban in I. Bezprozvanny laboratory. This work led to a crystal structure of apo Grb2 N-SH3 (PDB ID 6SDF). However, our efforts to crystallize SH3:Sos1-X′ complex proved unsuccessful.

An alternative would be to obtain NMR structure of the covalent complex. However, as already pointed out, NMR structures are generally less accurate than their crystallographic counterparts^56^. In particular, it may be difficult to reliably detect the repositioning of several residues in the n-Src loop by ca. 1–2 Å based on NMR coordinates. Moreover, standard structure-solving methods would require isotopic labeling of the (chemically modified) Sos1-X′ peptide, which is prohibitively expensive. Without the labeled peptide, it is difficult to obtain a good structural model of the flexible polyarginine tail and the thioether linker.

In the absence of high-quality structural data, we used NMR chemical shifts to validate the obtained SH3:Sos1-X′ model. This approach has been introduced following the development of structure-based methods for calculation of protein chemical shifts^57–61^. Like others, we use secondary chemical shifts, ${\delta }_{sec}=\delta -{\delta }_{rc}$, that are supposedly free from the effect of primary structure and sensitive only to higher-order (secondary, tertiary, etc.) structure. Note that our definition of the secondary shift is analogous to the one reported originally^62,63^. The calculations were conducted using the chemical shift prediction program SPARTA+^64^. Specifically, MD frames were extracted from the SH3:Sos1-X′ trajectory with a step of 1 ns and processed by the program; the results were subsequently averaged and corrected for the random-coil shifts *δ*_*rc*_ as catalogued in SPARTA+. The experimental shifts were corrected using the same *δ*_*rc*_ values.

**Figure 6:**
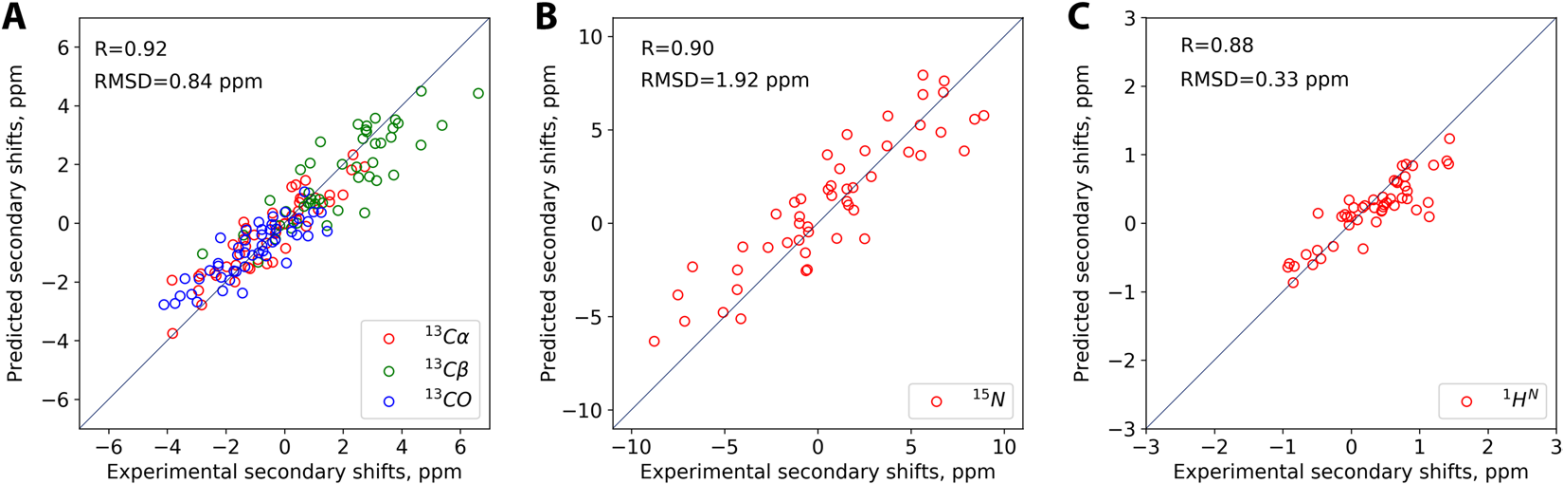
Correlation between the experimental and predicted secondary chemical shifts *δ*_*sec*_ in SH3:Sos1-X′ complex. The predicted values are from the MD model of SH3:Sos1-X′ developed in this work, which was processed using SPARTA+ software.

The correlation between the experimental and predicted (trajectory-based) *δ*_*sec*_ is illustrated in Fig. 6. The obtained correlation coefficients, 0.88–0.92, suggest that the model is in agreement with the experimental observations. In principle, chemical shifts are exquisitely sensitive to fine details of protein structure. However, their usefulness in this context is limited because of the relatively low accuracy of the available predictor programs. For instance, SPARTA+ claims the accuracy (i.e. *rmsd* between the predicted and experimentally measured shifts) of 1.09, 0.94 and 1.14 ppm for ^13^C′, ^13^C^α^ and ^13^C^β^ spins, respectively. In our analysis, we have obtained the *rmsd* of 0.84 ppm for the combined data from these carbon spins, Fig. 6A. A significant uncertainty margin associated with chemical shift predictions essentially limits the “resolution” of this method as a structure-validation tool (in other words, *δ*_*sec*_ *rmsd* is not nearly as informative as, for example, crystallographic *R*_*free*_^65^).

To advance further, we choose to compare the results in Fig. 6 with other similar analyses. As a first step, we used the NMR structure of noncovalent complex SH3·Sos1-X′ (1GBQ) as a model for covalent complex SH3:Sos1-X′. This is not unreasonable, given that peptide conjugation leads only to minimal structural changes in the complex, see Fig. 5. Moreover, one may expect that the experimentally determined structure (SH3·Sos1-X′) is in certain ways superior to the MD-based model (SH3:Sos1-X′). However, the results have not borne out these expectations. 1GBQ proved to be a markedly worse model of the covalent complex, as indicated by lower Pearson coefficients and significantly increased *rmsd*s: 1.05 vs. 0.84 ppm for ^13^C, 2.71 vs. 1.92 ppm for ^15^N, and 0.53 vs. 0.33 ppm for ^1^H^N^ spins (cf. Figs. 6 and S8). This outcome reflects favorably on our MD-based model of SH3:Sos1-X′. Specifically, it suggests that our model is not simply a mechanically altered version of 1GBQ, but rather a distinctly better representation of the actual covalent complex.

It is also instructive to compare the results in Fig. 6 with benchmarks from one of the popular model proteins, such as ubiquitin. To this end, we correlated the experimental chemical shifts of ubiquitin with SPARTA+ predictions using the crystallographic structure 1UBQ^66^. It is anticipated that this example should yield the best correlation obtainable with SPARTA+ software. Indeed, SPARTA+ has been trained on high-resolution x-ray structures and, accordingly, produces best results when used with such structures^67^. Furthermore, 1UBQ was actually a part of the training set, which should additionally improve the quality of predictions. It should also be noted that 1UBQ has been extensively validated using various solution NMR data and proved to be a good model for ubiquitin in solution^68^. The results from 1UBQ calculations are presented in Fig. S9A–C. The obtained degree of correlation is comparable to the one in Fig. 6. Specifically, the Pearson coefficients are the same for ^13^C, *r* = 0.92 vs. 0.92, and nearly the same for ^15^N, *r* = 0.90 vs. 0.89, while for ^1^H^N^ our results using SH3:Sos1-X′ model are somewhat better, *r* = 0.88 vs. 0.80. In terms of *rmsd*, 1UBQ calculations show slightly better agreement for ^13^C (0.80 vs. 0.84 ppm) and ^15^N (1.87 vs. 1.92 ppm), while SH3:Sos1-X′ calculations prove a little more accurate for ^1^H^N^ (0.33 vs. 0.39 ppm). Of note, the latter result does not depend on proton optimization in the crystal structure of ubiquitin.

In addition to the crystallographic structure 1UBQ, we have also considered the recently reported ultra-accurate solution structure 2MJB^68^, as well as 1.23-μs MD trajectory of ubiquitin recorded in house under Amber ff14SB force field. The former led to chemical shift predictions on par with 1UBQ, see Fig. S9D–F. The latter produced the results that appeared to be slightly worse, Fig. S9G,H. This last observation is in agreement with the findings of Li and Brüschweiler, who concluded that the application of SPARTA+ and other similar predictors to MD trajectories do not lead to any improvement compared to high-resolution crystallographic structures and, in fact, tends to slightly degrade the accuracy of the predictions^67^.

In conclusion, the comparison with ubiquitin suggests that our SH3:Sos1-X′ model is of very good quality. As a template for chemical shift predictions, the SH3:Sos1-X′ model shows the same level of performance as a high-resolution x-ray structure, i.e. the highest level of performance attainable with SPARTA+ (as well as other empirical predictors). It is difficult, however, to make any more specific claims in this regard because of the limitations of the chemical-shift-based validation procedure.

## Concluding Remarks

It should be instructive to compare our approach with some of established MD-based modeling tools that, in principle, could be used to model peptide-protein conjugation. Of special interest are those methods that use empirical reactive force fields (ERFF), such as Empirical Valence Bond approach or Adiabatic Reactive Molecular Dynamics^19–22^.

In ERFF methods, it is normally assumed that both initial state of the system (i.e. in our case noncovalent complex) and its final state (covalent complex) are known. The main emphasis is on reconstruction of the potential energy surface on the path from the initial to the final state. This is achieved by means of *ab initio* calculations and may involve calibration against experimental data. For reactions with low barriers, ERFF methods can simulate the transition of the system from the initial to the final state^21^. More typically, however, the reactions of interest feature high barriers. In this case, ERFF methods rely on various biasing schemes, such as umbrella sampling^69^. Such approach allows one to estimate the magnitude of free-energy barrier Δ*G*, explore the details of transition state, and investigate the effect of protein environment on Δ*G*. All of this is the focus and the stated goal of the ERFF methods.

In our approach, we know the structure of the initial state (noncovalent complex), but not the final state (covalent complex). Unlike ERFF methods, we are not interested in the details of the transition state, but instead seek to build a realistic model of the final state. The reaction is initiated when the two reactive groups approach each other to within a certain minimum distance (which is a reasonable approximation for the starting point). The reaction is driven by switching the simulation from one potential energy surface to another. In principle, this approach is fraught with a risk of large perturbations to energy and temperature. However, in our implementation the switching is conducted over a relatively long period of time, as controlled by the appropriate switching function. Under these conditions, the excess energy is thermalized and successfully absorbed by the thermostat, thus avoiding any significant perturbations to the system. Previously, such switching functions have been employed in the context of different ERFF schemes^22,70^.

In summary, our method can be viewed as a modification of ERFF strategy, where we execute the transition from the initial to the final state with no interest in the details of the intervening barrier (cf. Fig. 4B). The adiabatic character of the transition is achieved by slow switching from one potential energy surface to the other. In this sense, our method can also be likened to a steered simulation. The distinction is that our algorithm is driven by structure-informed force field terms rather than a preselected geometric variable (e.g. certain characteristic distance)^71,72^.

While the transition state obtained in our method is broadly reasonable, we make no effort to reproduce the correct barrier height, etc. Consequently, our method has only limited ability to predict the efficiency of the reaction. Specifically, it can address steric factors controlling the approach of the reactive groups, but not the chemistry *per se*. At the same time, our method demonstrated its ability to produce a reasonable model for the final state (covalent complex), which is duly relaxed and structure-optimized under the appropriate force-field potential. Note that such model building is not a part of the original ERFF mandate.

In addition to ERFF methods, it is also worth mentioning an alternative modeling technique known as covalent docking^73^. In principle, this technique can also be used to build a model of covalent peptide-protein complex. However, in practice the existing programs can only handle small-molecule ligands and not peptidic ligands. Furthermore, this approach suffers from limitations that are common to docking programs, e.g. it restricts the flexibility of the system (treating the scaffold of the target protein as rigid). This makes it unsuitable for the purpose of the current study, where we seek to capture (subtle) structural changes caused by covalent bonding of the peptide ligand.

Of particular note, the described method evolves the system under the control of native, unaltered force field (with the exception of 2-ns period, representative of the chemical reaction). Accordingly, the speed of the simulations is essentially the same as in conventional simulations. The proposed MD protocol should be a useful complement to experimental studies of peptide-protein conjugates. First, MD modeling sheds light on the likelihood of the peptide reactive group making contact to its target residue on the surface of the protein. Many reactive groups can bond with more than one type of amino acid; some of these reactions are undesirable since they lead to unstable conjugates. MD modeling allows one to estimate the likelihood of generating such off-target products. This kind of *in silico* identification of possible conjugation sites can be of practical interest^15^. Second, our method permits building of high-quality models for peptide-protein conjugates. Such models can be helpful in numerous applications involving imaging, therapeutics, biotechnology, etc., especially when the conjugates do not easily lend themselves to structural characterization (such as the case with SH3:Sos1-X′). This is particularly true in situations where the initial models are incomplete, e.g. the coordinates of a flexible peptide tail or a protein loop are not accurately known (which is the case with SH3·Sos1-X′, where the peptide is partially disordered). In this situation, MD-based methods, such as described in this paper, can help to model the missing fragments and thus augment the existing structural data. Third, our method can offer some insight into stability of peptide-protein conjugates. Experimental results suggest that various covalent modifications can have a destabilizing effect on protein structure^48,74–76^. This has far-reaching implications for therapeutic applications of covalent peptidic ligands. It is anticipated that MD models can shed light on structural and dynamic origins of such effects.

## Materials and Methods

### Sample preparation

pET-28 vectors for Grb2 N-SH3 (WT and C32S) encoding residues 2–59 of human Grb2 have been purchased from GenScript. The protein additionally containing N-terminal His_6_-tag was expressed in Rosetta DE3 cells (Novagen) using minimal M9 media. ^15^N-NH_4_Cl and ^13^C-glucose supplements have been used for isotope labeling. Protein expression was induced when OD_600_ reached 0.8. After 16 hours of incubation at 37 °C, cells were harvested, suspended in the standard lysis buffer (containing 2 mM of DTT) and then homogenized by SPEX SamplePrep 6870 Freezer/Mill followed by three rounds of sonication on ice. The sample was purified using Ni-affinity and size-exclusion chromatography (columns GE HisTrap HP and GE Sephacryl S-200 HR). Sos1-X′ and Sos1-C peptides were synthesized by Pepmic Inc. The buffer composition was 20 mM sodium phosphate, 10 mM DTT, 10% D_2_O, 0.01% NaN_3_, pH 7.2 (unless indicated otherwise).

### NMR experiments

All measurements were conducted at 25 °C using 500 MHz Bruker Avance III spectrometer equipped with TBI room temperature probe. For spectral assignment of noncovalent (covalent) complex we used the sample containing 1.5 mM of ^15^N,^13^C-labeled protein and two-fold excess of Sos1-C (Sos1-X′) peptide. The buffer additionally contained 150 mM NaCl. The covalent complex was obtained by overnight incubation of Grb2 N-SH3 with Sos1-X′ at room temperature; the completion of the conjugation reaction was confirmed by SDS-PAGE. Spectral assignment was obtained using the standard suite of triple-resonance experiments: HNCO, HNCACB, HN(CA)CO and HN(CO)CA^77^; the results were found to be in agreement with the data of Wittekind *et al*.^18^.

Binding affinity of Sos1-X′ to C32S Grb2 N-SH3 has been determined using the sample with protein concentration 100 μM. The peptide concentration was incremented from 0 to 160 μM in a step of 20 μM and then brought to 240 μM. The spectra were acquired using ^1^H,^15^N-BEST-HSQC sequence^78^. The results were analyzed using the program TITAN^39^. Conjugation kinetics was investigated using the sample containing 1 mM Grb2 N-SH3. Following the addition of 2 mM Sos1-X′, a series of back-to-back ^1^H,^15^N-BEST-HSQC experiments was recorded (24 mins per spectrum). Peak volumes were obtained using nlinLS^79^ and then fitted assuming pseudo first order (exponential) kinetics.

The refolding of noncovalent complex was investigated using the sample containing 100 μM Grb2-N SH3 and 300 μM Sos1-C. Covalent complex was obtained by overnight incubation of 100 μM of Grb2-N SH3 with 300 μM Sos1-X′. The buffer additionally contained 150 mM NaCl. The samples were denatured by 30-min incubation at 70 or 90 °C and then returned to room temperature. ^1^H,^15^N-BEST-HSQC spectra were recorded before and after this procedure.

### SDS-PAGE

The freshly prepared protein material was reduced by 10 mM DTT, divided into several portions and transferred to 50 mM sodium acetate buffer (pH 4.0 and 5.0), 50 mM sodium phosphate buffer (pH 6.0 and 7.0) and 50 mM Tris-HCl buffer (pH 8.0). The obtained samples, containing 100 μM Grb2 N-SH3 and 1 mM DTT, were incubated for 20 mins with 200 μM Sos1-X′ and then loaded on non-reducing tris-glycine 14% acrylamide gel as is (i.e. without quenching the reaction or boiling). The gel was stained using Coomassie Blue.

### LC-MS/MS analysis

Samples of apo Grb2 N-SH3 and Grb2 N-SH3 conjugated with Sos1-X′ were dissolved in 50 mM ammonium bicarbonate buffer and digested with trypsin (10 ng/μl) for 16 h at 37 °C. The reaction was stopped with 0.5% formic acid. The obtained tryptic peptide fractions (injection volume 2 uL) were analyzed in triplicates on a nano-HPLC Agilent 1100 system (Agilent Technologies, Inc.) coupled to a 7 T LTQ-FT Ultra mass-spectrometer (Thermo Electron Bremen GmbH) using a nanospray ion source, as described previously^80,81^. HPLC separation was performed on a capillary column packed in-house (75 μm i.d. × 12 cm fused silica capillary filled with Reprosil-Pur Basic C18, 3 μm/100 Å, Dr. Maisch HPLC GmbH). Gradient elution was carried out at a flow rate of 0.3 μL/min with the mobile phase A being 0.1% formic acid in water and mobile phase B – 0.1% formic acid in acetonitrile. After pre-equilibration with 3% (v/v) solvent B, a 30 min linear gradient from 3% to 50% was applied, followed by a 5 min gradient from 50% to 90% and then a 10 min isocratic elution with 90% solvent B. MS and MS/MS data were obtained in data-dependent mode using Xcalibur (Thermo Finnigan LLC) software. The precursor ion scan MS spectra (m/z range 300–1600) were acquired in the FTICR cell with resolution R = 50,000 at m/z 400 (number of accumulated ions: 5·10^6^). Five most intense ions from each parent scan were isolated and fragmented by collision-induced dissociation (CID), electron-capture dissociation (ECD) or ECD with in-source decay. Dynamic exclusion was used with 30 s duration^80,81^.

MS data were searched using PEAKS Studio 8.5 software against the SwissProt human database (augmented with user-supplied sequences of the expected tryptic peptides). The initial mass tolerance was set to 50 ppm for full scans and 0.1 Da for MS/MS. Most common natural or artifactual modifications, such as oxidation of methionine, histidine and tryptophan, deamidation of asparagine and glutamine, and acetylation of N-terminus, were used as variable modifications in the database search. In addition, possible preparation-related modifications, such as chloroacetylation of lysine and DTT modifications of cysteine, were included in the search. Finally, the expected target modification, i.e. modification of cysteine with acetyllysine, was also added. Separately, a modification search with a wide range of possible modifications has been carried out, allowing for up to five variable modifications per peptide. The cutoff false discovery rate was set to 0.1%. At least one unique peptide identification per protein was required. Using the described LC-MS/MS protocol, the target modification was identified in the SH3:Sos1-X′ sample, but not in the control apo sample.

### Parameterization of non-native residues for Amber ff14SB force field

We have obtained force field parameters for several small molecules and modified amino-acid residues relevant for the problem at hand. To this end, we have used the following standard algorithm. First, appropriate small-molecule models were chosen and geometry-optimized using B3LYP functional^82,83^ with 6–31 G(d) basis set^84,85^. Second, electrostatic potential of the optimized models was calculated using Hartree-Fock method with the same basis set^86^. Third, the corresponding electrostatic potential (ESP) atomic charges^87^ and restrained electrostatic potential (RESP) atomic charges^88^ were derived using the RESP module in Amber 16. Based on the similarity of ESP charges, we transfer (previously reported or newly calculated) RESP charges from one molecule to another. Fourth, other necessary parameters that are absent from the standard ff14SB force field have been adopted from GAFF2^51,89^. All quantum chemical calculations in the above procedure have been performed using Gaussian^90^.

Small-molecule models and amino-acid residues that have been treated in this manner are listed below. The chemical structures of the amino-acid residues and the respective atomic charges are summarized in Fig. S10.

(i) Non-terminal deprotonated lysine (LYN). Used for method calibration (the corresponding force-field parameters are available in ff14SB).
(ii) C-terminal deprotonated lysine (CLYN). Atomic charges for side-chain atoms beginning with δ-methylene group are the same as in LYN.
(iii) 2-chloro-N-propylacetamide. A partial model for N^ε^-chloroacetyl lysine side chain.
(iv) Non-terminal N^ε^-chloroacetyl lysine (LYC). Atomic charges for backbone atoms, as well as side-chain atoms up to and including γ-methylene group, are the same as in LYN. Atomic charges for side-chain atoms beginning with δ-methylene group are the same as RESP charges from 2-chloro-N-propylacetamide.
(v) C-terminal N^ε^-chloroacetyl lysine (CLYC). Atomic charges for backbone atoms, as well as side-chain atoms up to and including γ-methylene group, are the same as in CLYN. Atomic charges for side-chain atoms beginning with δ-methylene group are the same as RESP charges from 2-chloro-N-propylacetamide.
(vi) S-(2-propylamino-2-oxoethyl)-cysteine capped with acetyl and N-methylamide in N- and C-terminal positions, respectively. A partial model for the cysteine residue conjugated to N^ε^-chloroacetyl lysine.
(vii) Non-terminal cysteine residue modified by conjugation with N^ε^-chloroacetyl lysine (CYZ). Atomic charges for N, H^N^, C and O atoms are the same as in CYS. Other atomic charges are the same as RESP charges from S-(2-propylamino-2-oxoethyl)-cysteine.
(viii) Non-terminal N^ε^-chloroacetyl lysine residue modified by conjugation with cysteine (LYZ). Atomic charges for backbone atoms, as well as side-chain atoms up to and including γ-methylene group, are the same as in LYN. Other atomic charges are the same as RESP charges from S-(2-propylamino-2-oxoethyl)-cysteine. The boundary between LYZ and CYZ residues is across the C^θ^-S^γ^ bond.
(ix) C-terminal N^ε^-chloroacetyl lysine residue modified by conjugation with cysteine (CLYZ). Atomic charges for backbone atoms, as well as side-chain atoms up to and including γ-methylene group, are the same as in CLYN. Other atomic charges are the same as RESP charges from S-(2-propylamino-2-oxoethyl)-cysteine. The boundary between CLYZ and CYZ residues runs across the C^θ^-S^γ^ bond.

Note that entries (*vii*-*ix*) do not involve any new calculations compared to (*i*-*vi*). Of the above amino-acid residues, CLYC, CLYZ and CYZ have been used in our MD simulations of Sos1-X′ conjugation with Grb2 N-SH3.

### MD simulations

Initial coordinates to simulate the complex of Grb2 N-SH3 with Sos1-X′ were from PDB ID 1GBQ. To bring the structure in line with our experimental design, we appended N^ε^-chloroacetyl lysine residue (X′, CLYC) to the C-terminus of the Sos1 peptide and treated the reactive cysteine residue C32 in the Grb2 N-SH3 as a thiolate anion (CYM), cf. the discussion of Fig. 2D. The protonation state of the system in the absence of histidine residues was standard for the target pH 7.2 (verified by PROPKA^91^ calculations). The structure was solvated with TIP3P water by building a truncated octahedron box with minimal 10 Å separation between the protein/peptide atoms and the boundary. The solvated system was neutralized by adding a single Na^+^ ion, subjected to energy minimization (500 steps using harmonic restraints with force constant 200 kcal/mol·Å^2^, followed by 100 steps with no restraints), then heated from 0 to 293 K and equilibrated for 1 ns at 293 K. The subsequent simulations were conducted in Amber 16 under ff14SB force field (the nonstandard amino acid types added to the force field are described above, alterations to the force field during the 2-ns reaction period are described below). The simulations were carried out in the NPT ensemble using Langevin thermostat with collision frequency 2 ps^−1^. A cutoff of 10.5 Å was used for (nonbonded) van der Waals and short-range electrostatic interactions; long-range electrostatic interactions were treated using the particle mesh Ewald scheme. All bonds involving hydrogen atoms were constrained using SHAKE algorithm. The integration step was 2 fs. The production rate using workstations equipped with Tesla K40 m GPU cards was 110 ns/card/day.

A special master script, written in python, controls the entire simulation process (see Fig. S6). The initial 500-ns portion of each SH3·Sos1-X′ trajectory is recorded in a conventional fashion. After that, the script continues recording the trajectory in 1-ns segments. Atomic coordinates and velocities (restart files) are saved at 1-ps intervals. After the segment is completed, the script loops over the frames and extracts the distances between C^θ^ (X′) and S^γ^ (C32) atoms. If during 1-ns time interval the distance never drops below 3.3 Å, the simulation is continued and the next 1-ns fragment is recorded. Otherwise, if the frame *n* is found where this distance is lower than 3.3 Å, the algorithm makes a decision on whether to initiate the reaction. This decision is made using the rejection sampling strategy with *p*_0_ set to 0.1 (i.e. the decision is random with the probability of positive outcome 0.1)^92^. If the outcome is negative, the scanning of the frames continues. Otherwise, the restart file *n* is invoked; the state of the system stored in this file is taken to be the initial state of the reaction.

During the subsequent reaction period T_*rxn*_ = 2 ns we use the force-field parameters interpolated between the initial state of the system, SH3·Sos1-X′, and its final state, SH3:Sos1-X′. This concept is illustrated in Fig. 3 for the force constants associated with C^θ^-Cl bond (decremented from its nominal value in the chloroacetyl group to zero) and C^θ^-S^γ^ bond (incremented from zero to its nominal value in the thioether linkage). The list of parameters subject to interpolation is given in Table S1 (the key to this table is shown in Fig. S11). The information on atomic charges is provided separately in Fig. S10.

The interpolation procedure is implemented as follows. The 2-ns period is divided into 10-ps intervals. At the beginning of each interval, the relevant force constants are incremented/decremented and the temporary parameter-topology (prmtop) file is created. The 10-ps trajectory segment is then recorded under the control of this temporary prmtop file. At the end of the T_*rxn*_ period, the temporary prmtop file is strictly equivalent to the generic prmtop file for the SH3:Sos1-X′ complex under ff44SB force field (augmented with the parameters for CYZ and CLYZ residues). Therefore, the simulation can be seamlessly continued further in a fully conventional manner. In doing so, we record 500 ns of such conventional trajectory representing the covalent SH3:Sos1-X′ complex.

The above procedure involves one technicality, which deserves a separate comment. For example, consider the pair of atoms C^θ^ and Cl that are initially bonded in the SH3·Sos1-X′ complex and then become separated in SH3:Sos1-X′ (chlorine atom moves into solvent in a form of Cl^–^ ion). Amber convention is such that van der Waals and electrostatic interactions between the two bonded atoms are nullified. This makes it impossible to implement the interpolation strategy for these two interactions. To circumvent this problem, we disable the C^θ^-Cl bond and replace it with an equivalent harmonic NMR restraint. Then we redefine C^θ^ and Cl as 1–4 atoms (by introducing a fictitious C^θ^-X-Y-Cl angle). The van der Waals and electrostatic interactions are allowed between 1–4 atoms and the corresponding force constants can be edited using ParmEd facilities^93^. This workaround has been used for several energy terms. Specifically, during the T_*rxn*_ period we disabled the original potentials associated with C^θ^-Cl and C^θ^-S^γ^ bonds, as well as C^β^-S^γ^-C^θ^, Cl-C^θ^-C^η^, Cl-C^θ^-H^θ1^, Cl-C^θ^-H^θ2^, Cl-C^θ^-S^γ^, S^γ^-C^θ^-C^η^, S^γ^-C^θ^-H^θ1^ and S^γ^-C^θ^-H^θ2^ angles, and replaced them with the appropriate artificial restraints. At the end of the T_*rxn*_ period, all these restraints were seamlessly replaced with the equivalent default potentials.

In an alternative version of this protocol, we used sigmoidal functions instead of linear functions to interpolate between the two sets of the force field parameters^22^. The results were unchanged (not shown).

MD trajectories were analyzed using the program PYTRAJ^94^, as well as PYXMOLPP2 package written in-house. Chemical shifts of Grb2 N-SH3 domain in SH3:Sos1-X′ complex were calculated on per-frame basis using the program SPARTA+^64^. For this purpose we have used the final 450 ns of each trajectory sampled with the step of 1 ns. The calculated shifts were averaged over 450 × 11 = 4,950 frames from eleven trajectories. To calibrate chemical shift calculations, we also used a separate 1.23-μs trajectory of ubiquitin. This simulation was conducted in the NVE ensemble using TIP4P-Ew water model^95^.

## Supplementary information

Supplementary Information

Supplementary Information 2

## Data Availability

Leap library files for all modified residues described in this paper, force-field parameter modification file and python script to run reactive MD simulations have been deposited to github.com/bionmr-spbu-projects/2019-GRB2-Sos1. The latter script is dependent on MD-control library PYRUN, which is available from github.com/bionmr-spbu/pyrun. PYXMOLPP2 package to process MD trajectories can be downloaded from github.com/bionmr-spbu/pyxmolpp2. The backbone NMR assignments of Grb2 N-SH3 have been deposited in BMRB under accession numbers 50104 (noncovalent complex with Sos1-C) and 50106 (covalent complex with Sos1-X′). All other data reported in this paper are available from the authors upon request.
