# Supplementary material for: Molecular Dynamics model of peptide-protein conjugation: case study of covalent complex between Sos1 peptide and N-terminal SH3 domain from Grb2
Source: Sci Rep. 2019 Dec 27;9:20219. doi: 10.1038/s41598-019-56078-7 (PMC6934455; doi:10.1038/s41598-019-56078-7)
Supplement: Supplementary file 1 — Supplementary Information [file 41598_2019_56078_MOESM1_ESM.pdf]

SUPPLEMENTARY INFORMATION

Molecular Dynamics model of peptide-protein conjugation: case study of covalent complex between Sos1 peptide and N-terminal SH3 domain from Grb2

Dmitry A. Luzik,[1†] Olga N. Rogacheva,[1,2†] Sergei A. Izmailov,[1]
Maria I. Indeykina,[3] Alexei S. Kononikhin,[3,4]
Nikolai R. Skrynnikov[1,5*]

[1] Laboratory of Biomolecular NMR, St. Petersburg State University, St. Petersburg 199034, Russia.

[2] Department of General Pathology, Institute of Experimental Medicine, St. Petersburg 197376, Russia

[3] N.M. Emanuel Institute of Biochemical Physics, Russian Academy of Sciences, Moscow 119991, Russia

[4] Laboratory of mass spectrometry, CDISE, Skolkovo Institute of Science and Technology, 121205 Moscow, Russia

[5] Department of Chemistry, Purdue University, West Lafayette, IN 47907, USA.

† These authors contributed equally: Dmitry A. Luzik and Olga N. Rogacheva

* Corresponding author. E-mail: nikolai@purdue.edu. Phone: (765)-494 8519

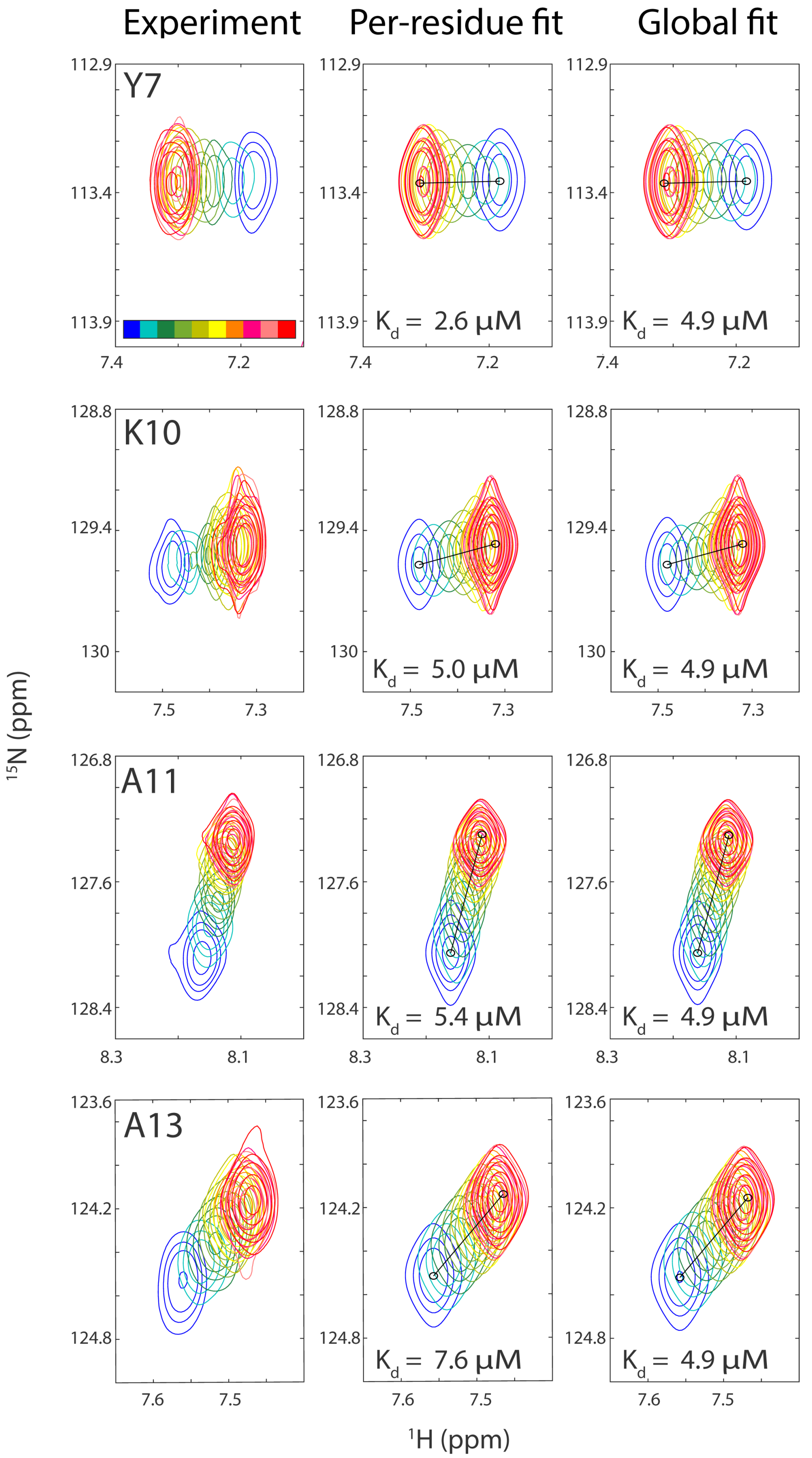

Figure S1. Continued on the next page.

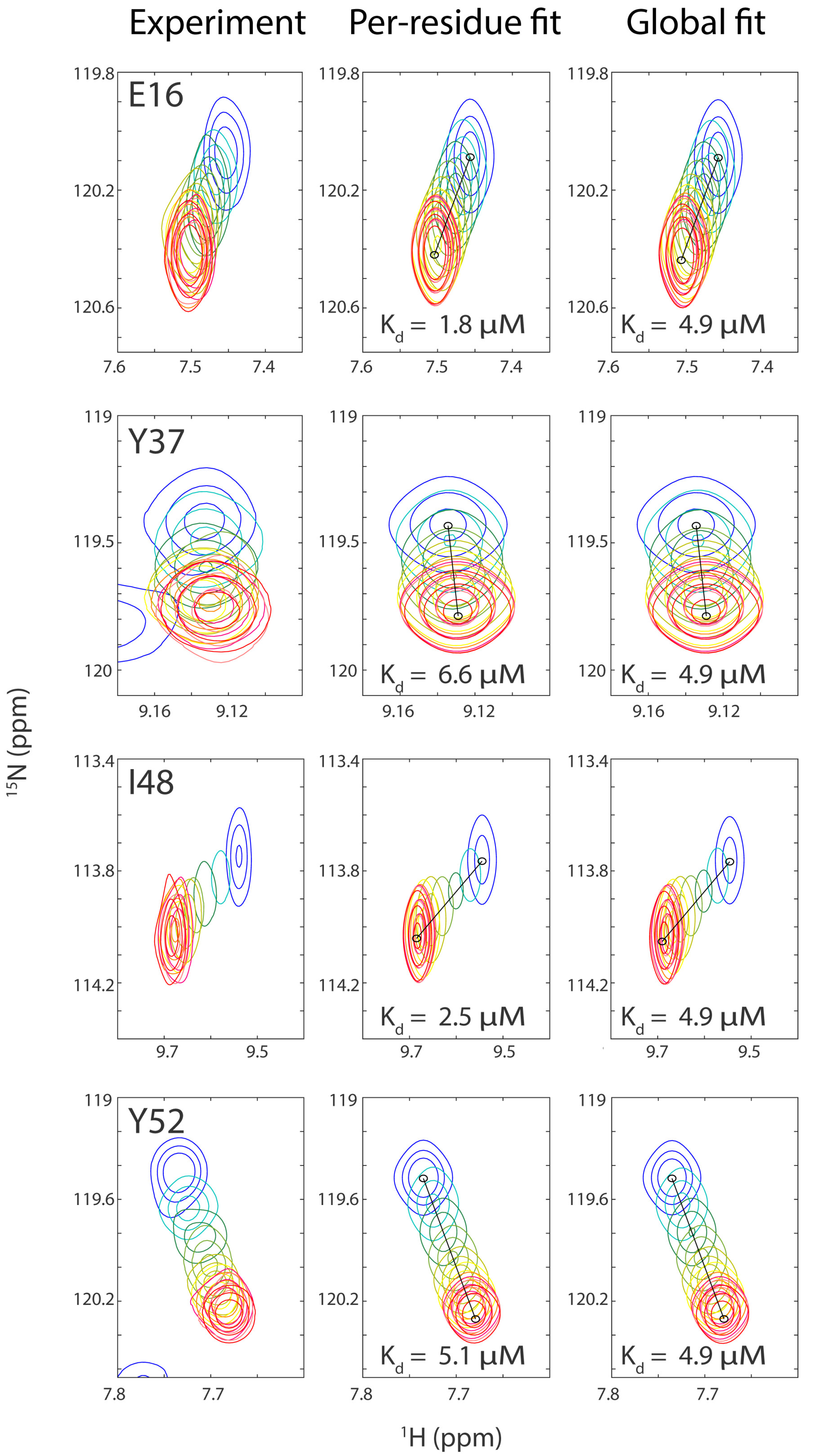

Figure S1. Observed and fitted $^{1}H^{N}$,^{15}N-HSQC titrations for eight residues in Grb2 N-SH3 C32S titrated with Sos1-X'. Color bar shown in the first panel codes the following series of peptide concentrations: 0 (blue), 20, 40, 60, 80, 100, 120, 140, 160 and 240 (red) μM. The initial (final) protein concentration is 100 (85) μM.

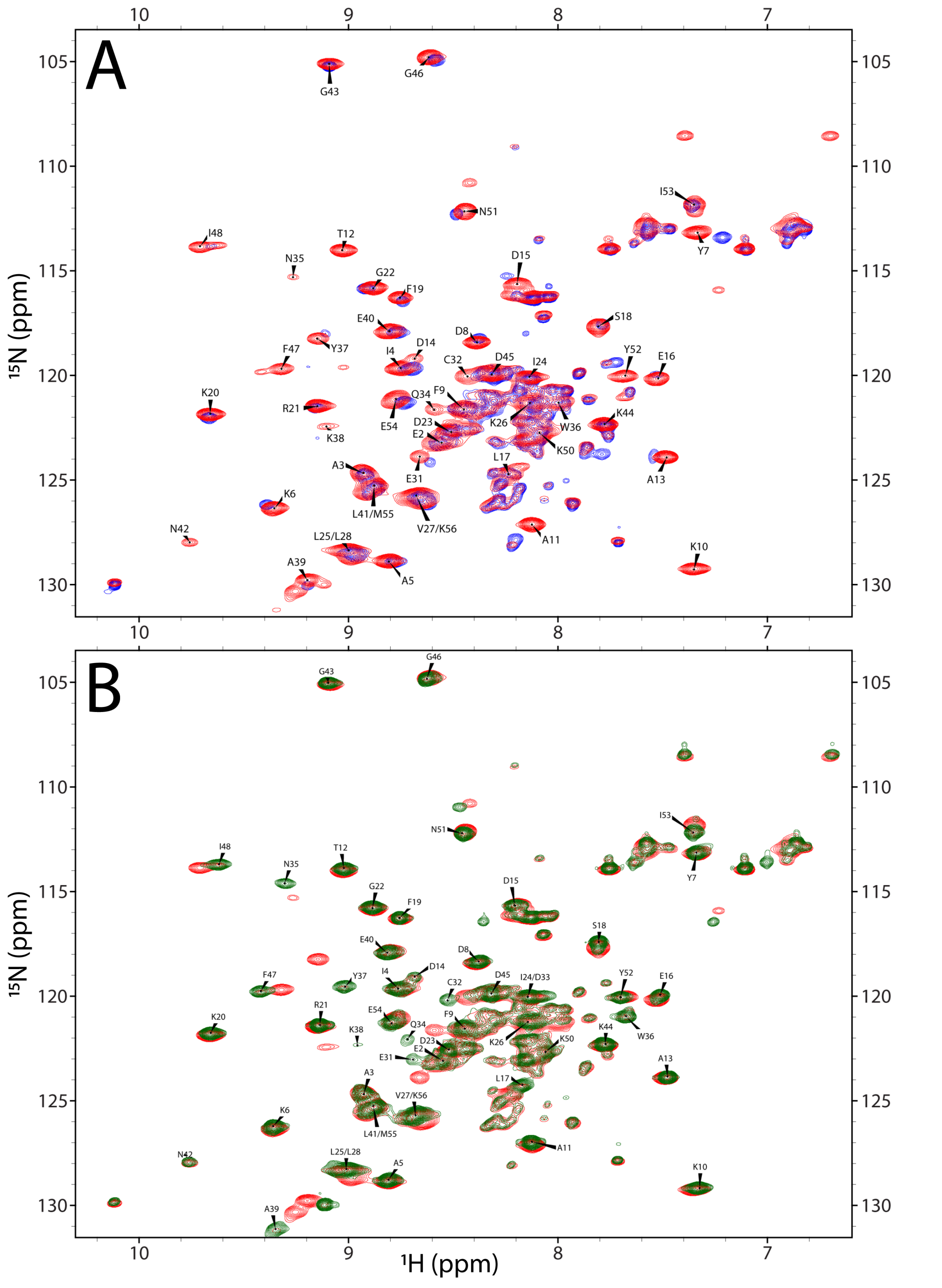

Figure S2. Superposition of $^{1}H^{N}$,^{15}N-HSQC spectra from (a) free Grb2 N-SH3 (blue) and noncovalent complex SH3·Sos1-X' (red); (b) noncovalent complex SH3·Sos1-X' (red) and covalent complex SH3:Sos1-X' (green). Protein concentration in all samples is 1 mM.

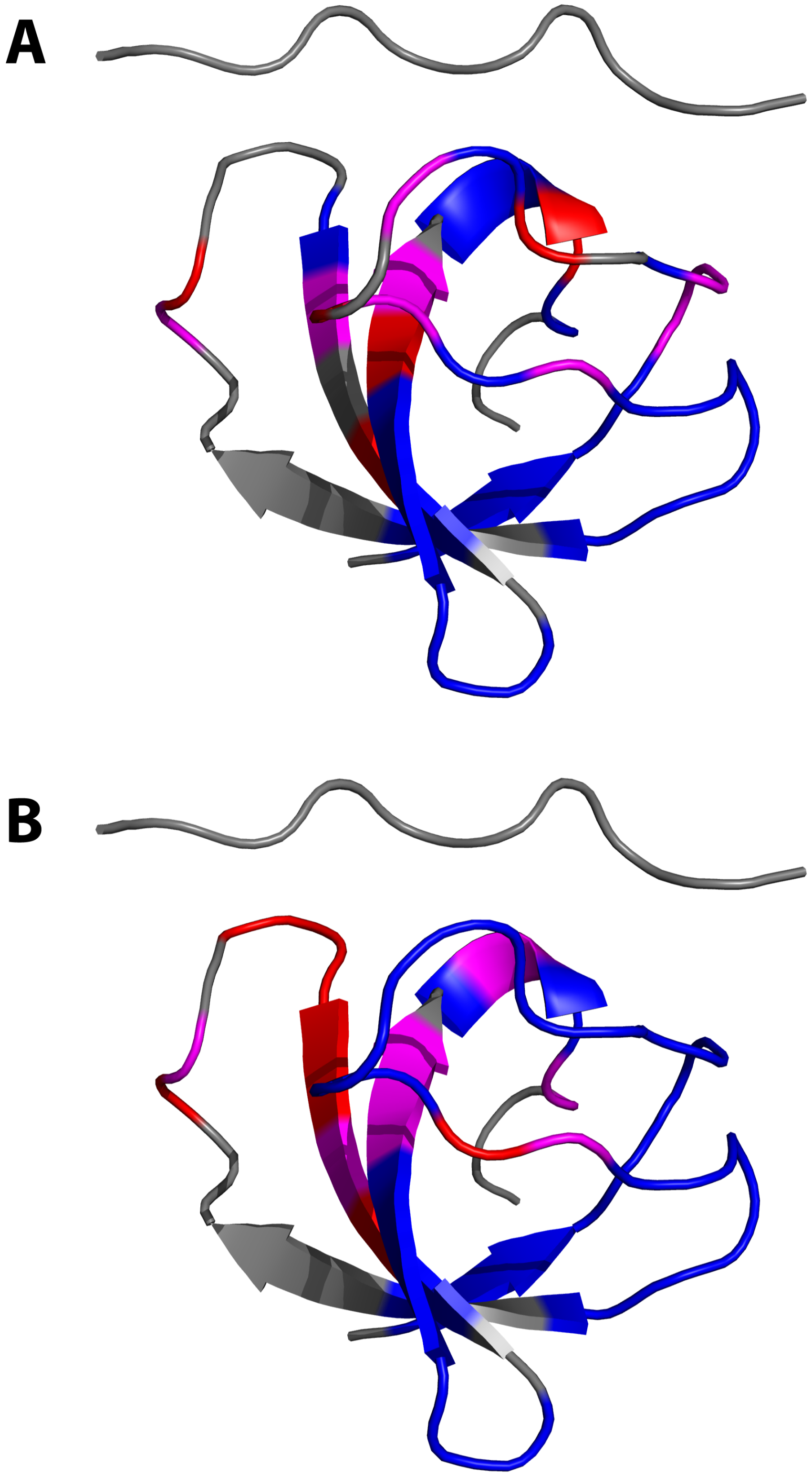

Figure S3. Mapping of the experimental chemical shift differences (a) Δ_{noncov} and (b) Δ_{cov} onto the coordinates 1GBQ. The data are from Fig. S2. Color-coding scheme: no data (spectral overlaps, prolines, etc.) – grey, $\Delta < 0.05$ ppm – blue, 0.05 ppm $< \Delta < 0.1$ ppm – magenta, $\Delta >$ 0.1 ppm – red.

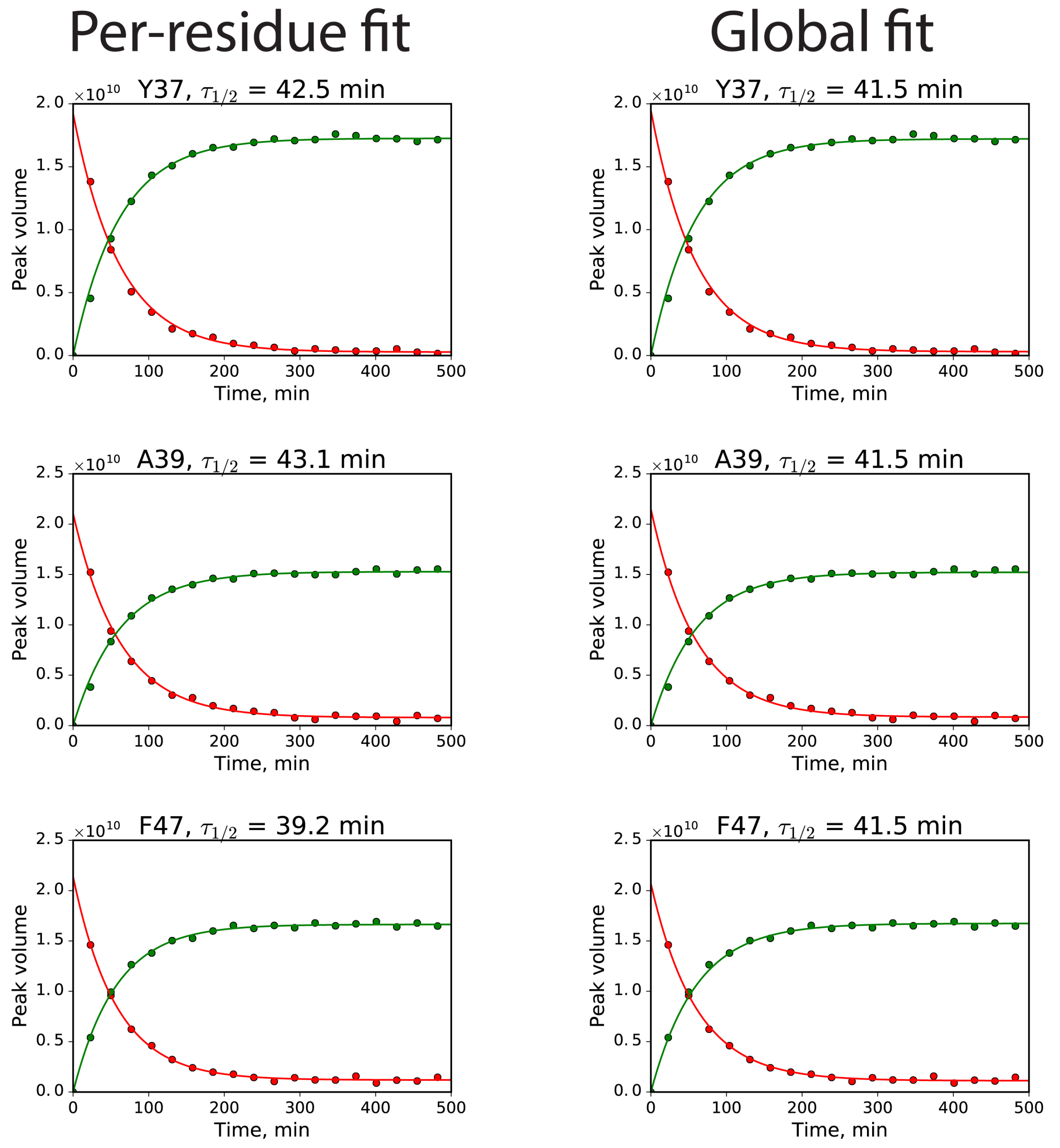

Figure S4. Kinetic curves for conjugation of Sos1-X' to Grb2 N-SH3. The reaction was monitored in real time using a series of back-to-back ^{1}H^{N},^{15}N-HSQC experiments. Shown are the volumes of spectral peaks from SH3·Sos1-X' and SH3:Sos1-X' species (red and green circles, respectively). Also shown are the exponential best-fit curves. Three residues were selected for this analysis that give rise to the well-resolved SH3·Sos1-X' and SH3:Sos1-X' peaks. The fitting has been conducted either on per-residue basis or, otherwise, using the global half-life parameter $\tau_{1/2}$ (left and right portions of the figure, respectively).

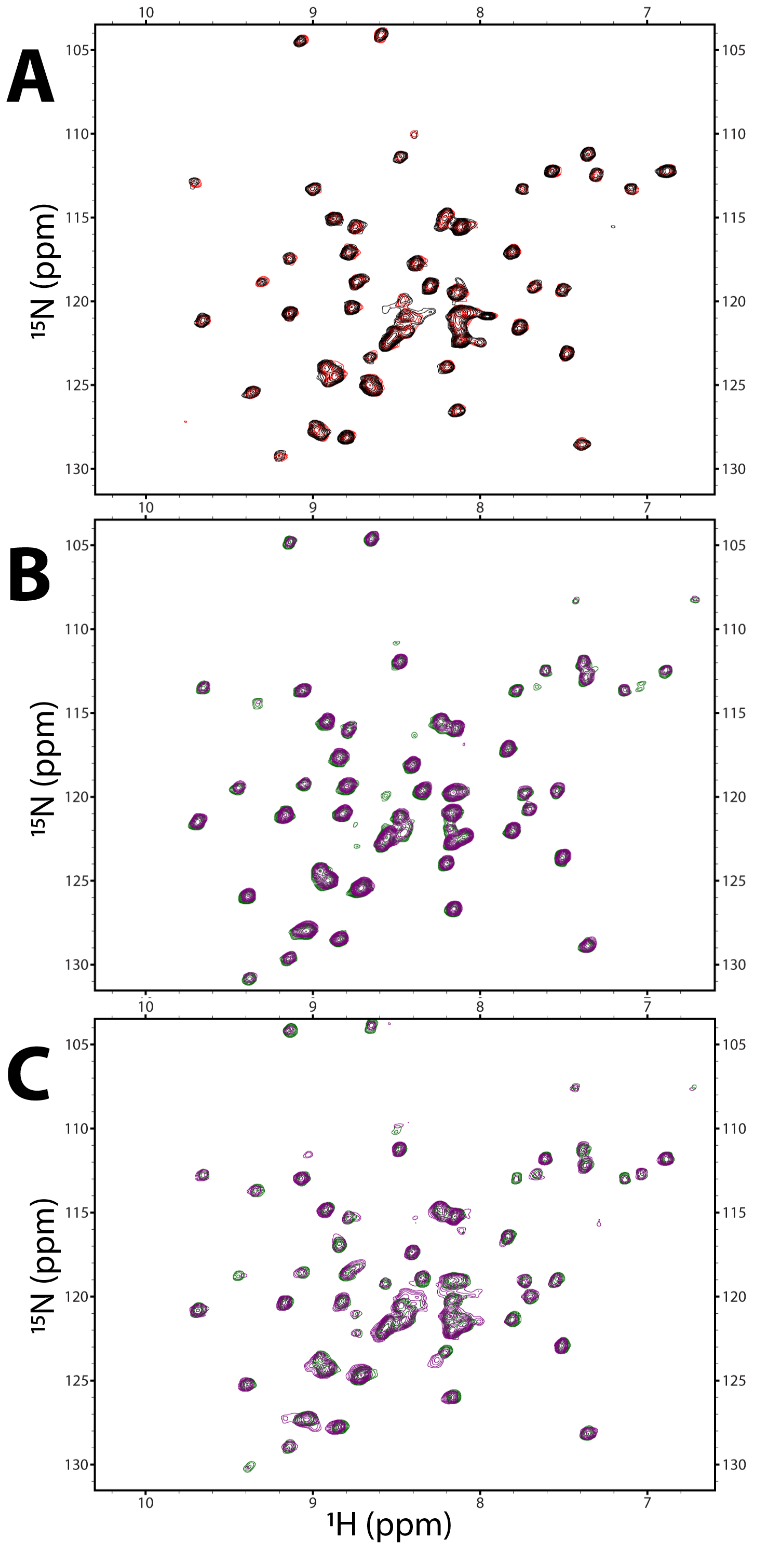

Figure S5. Superposition of $^{1}H^{N}$,^{15}N-HSQC spectra recorded before and after sample heating. (a) SH3·Sos1-C complex before and after 30 min incubation at 70 °C (red and black spectra, respectively). (b) SH3:Sos1-X' complex before and after 30 min incubation at 70 °C (green and purple spectra, respectively). (c) SH3:Sos1-X' complex before and after 30 min incubation at 90 °C (green and purple spectra, respectively).

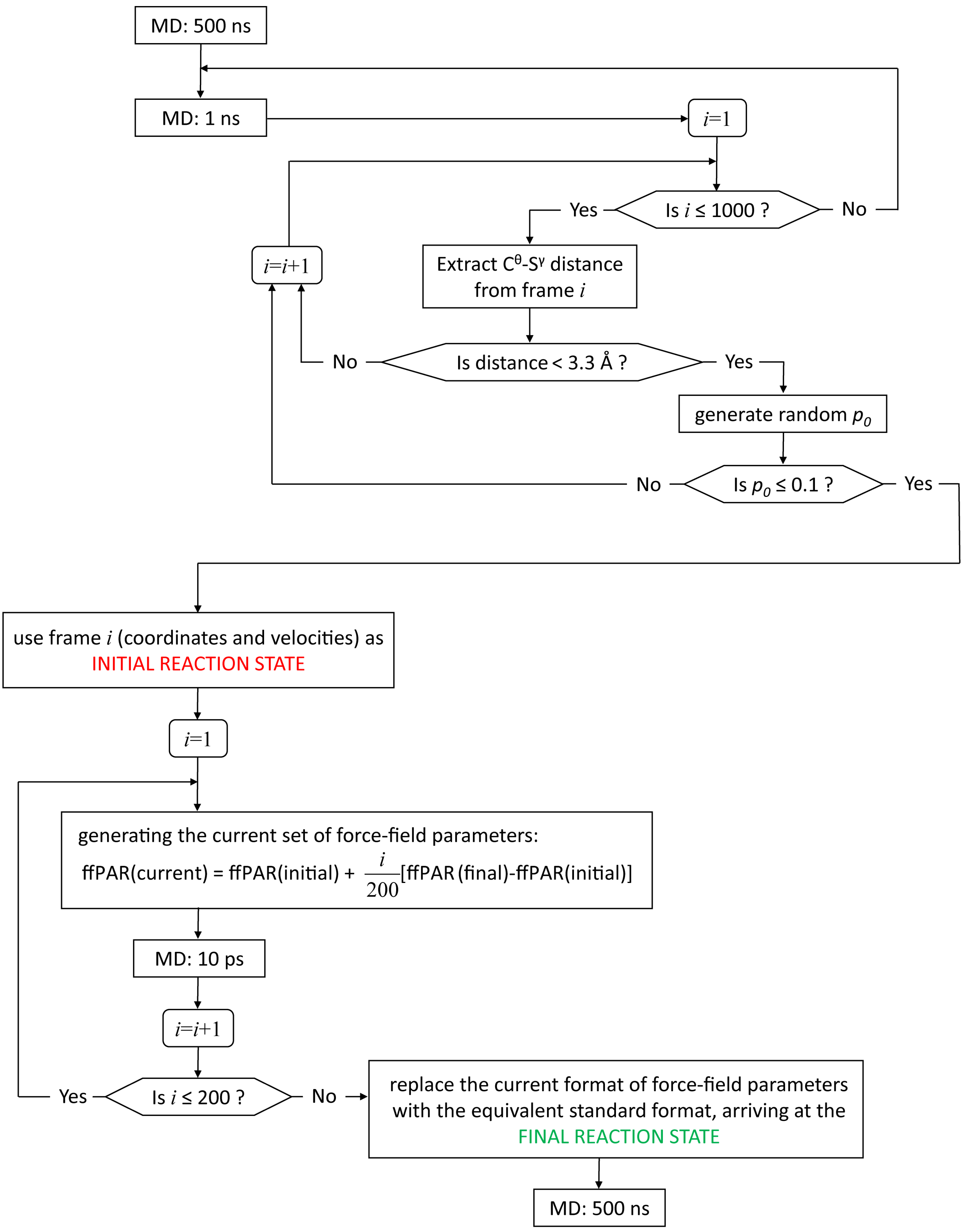

Figure S6. Schematic flowchart of the reactive MD simulation protocol used in this study.

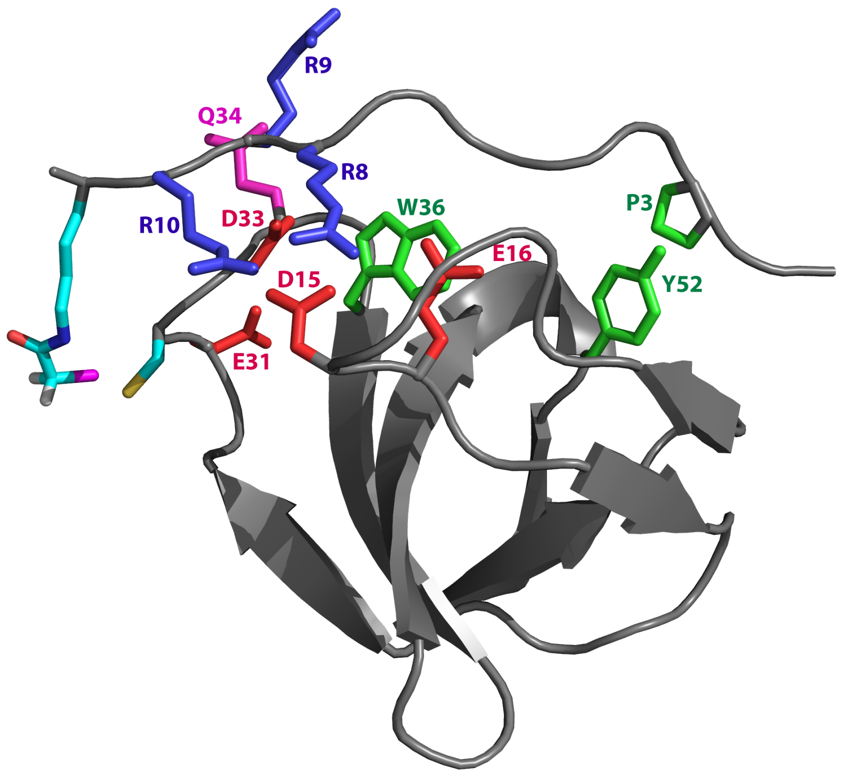

Figure S7. Non-covalent complex SH3·Sos1-X' and selected side chains relevant for the discussion in the text: (*blue*) positively charged Arg residues, (*red*) negatively charged Asp and Glu residues, (*green*) hydrophobic Pro, Trp and Tyr residues, (magenta) polar Gln residue. The structure and the visual perspective are the same as in Fig. 3b.

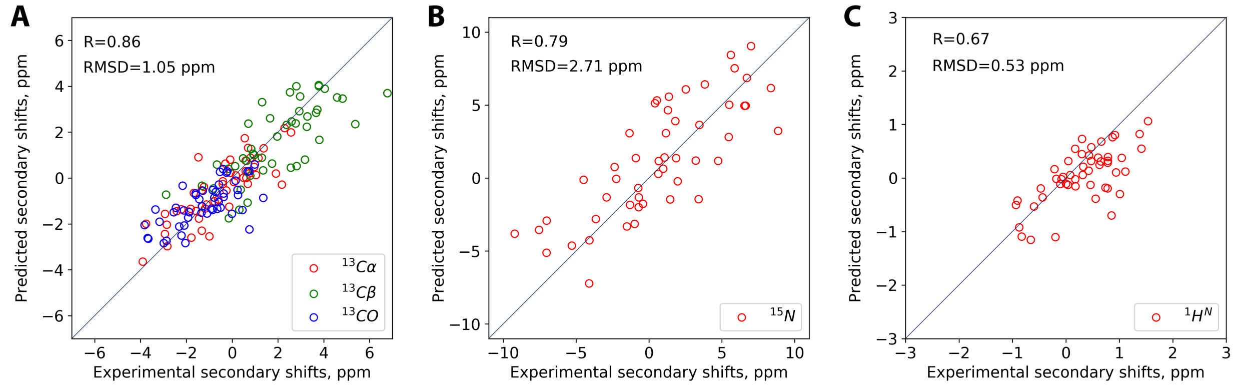

Figure S8. Correlation between the experimental and predicted secondary chemical shifts δ_{sec} in SH3:Sos1-X' complex, using NMR structure 1GBQ as an approximate model of covalent complex.

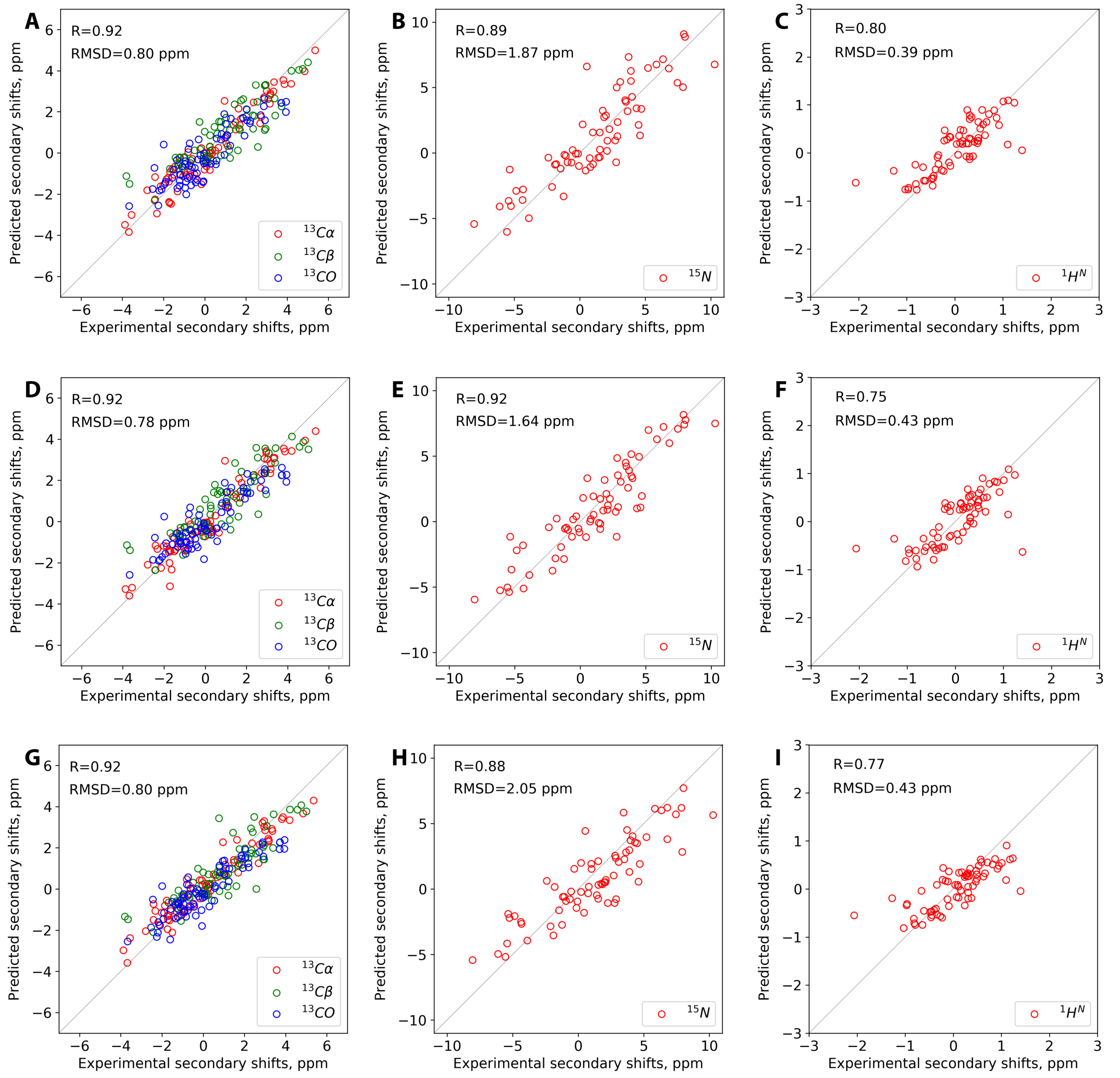

Figure S9. Correlations between the experimental and predicted secondary chemical shifts δ_{sec} in ubiquitin. The predicted shifts have been obtained by applying the program SPARTA+ to (a-c) crystallographic structure 1UBQ, (d-f) solution NMR structure 2MBJ, or (g-i) 1.23-μs MD trajectory of ubiquitin recorded under Amber ff14SB force field.

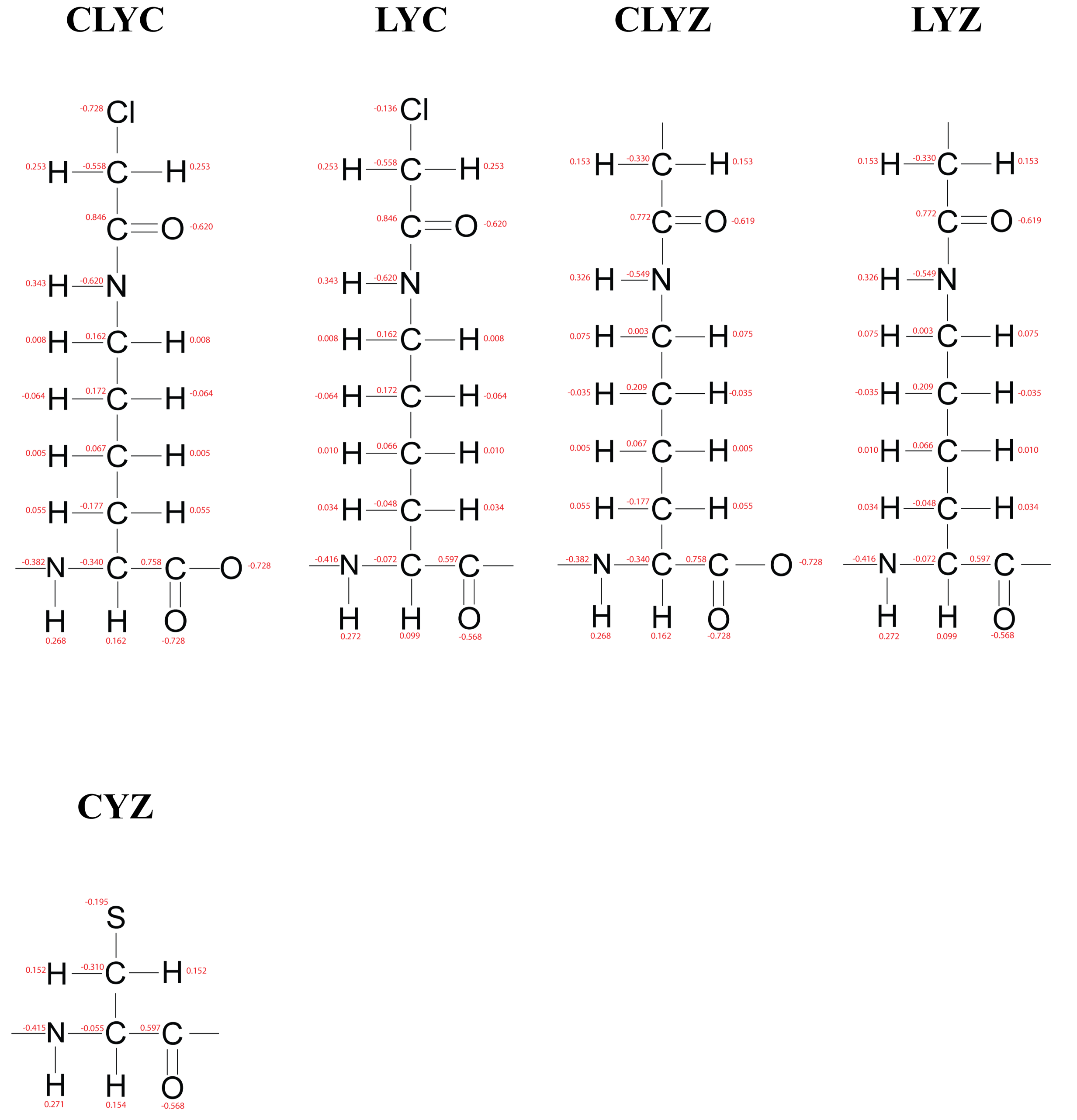

Figure S10. Chemical structure and atomic charges of new residue types defined in Amber ff14SB (see Materials and Methods).

Table S1. Force field parameters affected by conjugation of CYM to CLYC (LYC)

	Atom arrangements	Potential	Parameters before	Parameters after
1.	CL (Cl-)<br>Cl → Cl-	$U_{vdW}(r_{ij}) = \sqrt{\varepsilon_i \varepsilon_j}\left\{\left(\frac{R_{ij}}{r_{ij}}\right)^{12} - 2\left(\frac{R_{ij}}{r_{ij}}\right)^{6}\right\}$<br>$R_{ij} = R_i + R_j$	R_{CL}=1.948 Å	R_{Cl-}=2.513 Å
			ε_{CL}=0.265 kcal/mol	ε_{Cl-}=0.035591 kcal/mol
2.	SG<br>SH → S		R_{SG}=2.000 Å	R_{SG}=2.000 Å
			ε_{SG}=0.250 kcal/mol	ε_{SG}=0.250 kcal/mol
3.	CB-SG<br>CT-SH → 2C-S	$U(r_{ij}) = K_r\left(r_{ij} - r_0\right)^2$	r_0=1.81 Å	r_0=1.81 Å
			K_r=237 kcal/mol/Å^2	K_r=227 kcal/mol/Å^2
4.	C2-CL<br>CT-Cl → ...		r_0=1.766 Å	r_0=1.766 Å
			K_r=232 kcal/mol/Å^2	K_r=0 kcal/mol/Å^2
5.	C2-SG<br>... → CT-S		r_0=1.81Å	r_0=1.81Å
			K_r=0 kcal/mol/Å^2	K_r=227 kcal/mol/Å^2
6.	CA-CB-SG<br>CX-CT-SH → CX-2C-S	$U(\phi) = K_\phi\left(\phi - \phi_0\right)^2$	ϕ_0=108.6°	ϕ_0=114.7°
			K_ϕ=50 kcal/mol/rad^2	K_ϕ=50 kcal/mol/rad^2
7.	HB2-CB-SG<br>H1-CT-SH → H1-2C-S		ϕ_0=109.5°	ϕ_0=109.5°
			K_ϕ=50 kcal/mol/rad^2	K_ϕ=50 kcal/mol/rad^2
8.	HB3-CB-SG<br>H1-CT-SH → H1-2C-S		ϕ_0=109.5°	ϕ_0=109.5°
			K_ϕ=50 kcal/mol/rad^2	K_ϕ=50 kcal/mol/rad^2
9.	C1-C2-CL<br>C-CT-Cl → ...		ϕ_0=110.41°	ϕ_0=110.41°
			K_ϕ=71.7 kcal/mol/rad^2	K_ϕ=0 kcal/mol/rad^2
10.	CL-C2-H1<br>Cl-CT-H1 → ...		ϕ_0=108.5°	ϕ_0=108.5°
			K_ϕ=50 kcal/mol/rad^2	K_ϕ=0 kcal/mol/rad^2
11.	CL-C2-H2<br>Cl-CT-H1 → ...		ϕ_0=108.5°	ϕ_0=108.5°
			K_ϕ=50 kcal/mol/rad^2	K_ϕ=0 kcal/mol/rad^2

12.	SG-C2-C1[(b)]<br>… → S-CT-C		ϕ_0=108.84°	ϕ_0=108.84°
			K_ϕ=0 kcal/mol/rad^2	K_ϕ=63.788 kcal/mol/rad^2
13.	SG-C2-H1<br>… → S-CT-H1		ϕ_0=109.5°	ϕ_0=109.5°
			K_ϕ=0 kcal/mol/rad^2	K_ϕ=50 kcal/mol/rad^2
14.	SG-C2-H2<br>… → S-CT-H1		ϕ_0=109.5°	ϕ_0=109.5°
			K_ϕ=0 kcal/mol/rad^2	K_ϕ=50 kcal/mol/rad^2
15.	CB-SG-C2<br>… → 2C-S-CT		ϕ_0=98.9°	ϕ_0=98.9°
			K_ϕ=0 kcal/mol/rad^2	K_ϕ=62 kcal/mol/rad^2
16.	C-CA-CB-SG<br>C-CX-CT-SH → C-CX-2C-S	$U(\theta)=K_\theta\left\{1+\cos\left(n\theta-\theta_0\right)\right\}$	K_θ=0.1556 kcal/mol, n=3, θ_0=0°	K_θ=0 kcal/mol, n=3, θ_0=0°
			K_θ=0 kcal/mol, n=1, θ_0=0°	K_θ=0.602 kcal/mol, n=1, θ_0=0°
			K_θ=0 kcal/mol, n=2, θ_0=180°	K_θ=0.394 kcal/mol, n=2, θ_0 =180°
			K_θ=0 kcal/mol, n=3, θ_0=0°	K_θ=0.323 kcal/mol , n=3, θ_0=0°
			K_θ=0 kcal/mol, n=4, θ_0=0°	K_θ=0.278 kcal/mol, n=4, θ_0=0°
17.	N-CA-CB-SG<br>N-CX-CT-SH → N-CX-2C-S		K_θ=0.1556 kcal/mol, n=3, θ_0=0°	K_θ=0 kcal/mol, n=3, θ_0=0°
			K_θ=0 kcal/mol, n=1, θ_0=0°	K_θ=0.469 kcal/mol, n=1, θ_0=0°
			K_θ=0 kcal/mol, n=2, θ_0=180°	K_θ=0.021 kcal/mol, n=2, θ_0=180°
			K_θ=0 kcal/mol, n=3, θ_0=0°	K_θ=0.323 kcal/mol, n=3, θ_0=0°
			K_θ=0 kcal/mol, n=4, θ=0°	K_θ=0.064 kcal/mol, n=4, θ=0°
18.	HA-CA-CB-SG		K_θ=0.1556 kcal/mol,	K_θ=0.1556 kcal/mol,

	H1-CX-CT-SH → H1-CX-2C-S		n=3, θ_0=0°	n=3, θ_0=0°
19.	HB2-CB-SG-C2<br>... → H1-2C-S-CT		K_θ=0 kcal/mol,<br>n=3, θ_0=0°	K_θ=0.3333 kcal/mol,<br>n=3, θ_0=0°
20.	HB3-CB-SG-C2<br>... → H1-2C-S-CT		K_θ=0 kcal/mol,<br>n=3, θ_0=0°	K_θ=0.3333 kcal/mol,<br>n=3, θ_0=0°
21.	CA-CB-SG-C2<br>... → CX-2C-S-CT		K_θ=0 kcal/mol,<br>n=3, θ_0=0°	K_θ=0.3333 kcal/mol,<br>n=3, θ_0=0°
22.	CB-SG-C2-H1<br>... → 2C-S-CT-H1		K_θ=0 kcal/mol,<br>n=3, θ_0=0°	K_θ=0.3333 kcal/mol,<br>n=3, θ_0=0°
23.	CB-SG-C2-H2<br>... → 2C-S-CT-H1		K_θ=0 kcal/mol,<br>n=3, θ_0=0°	K_θ=0.3333 kcal/mol,<br>n=3, θ_0=0°
24.	CB-SG-C2-C1<br>... → 2C-S-CT-C		K_θ=0 kcal/mol,<br>n=3, θ_0=0°	K_θ=0.3333 kcal/mol,<br>n=3, θ_0=0°
25.	SG-C2-C1-NZ<br>... → S-CT-C-N		K_θ=0 kcal/mol,<br>n=2, θ_0=0°	K_θ=0 kcal/mol,<br>n=2, θ_0=0°
26.	SG-C2-C1-OZ<br>... → S-CT-C-O		K_θ=0 kcal/mol,<br>n=2, θ_0=0°	K_θ=0 kcal/mol,<br>n=2, θ_0=0°
27.	CL-C2-C1-NZ<br>Cl-CT-C-N → ...		K_θ=0 kcal/mol,<br>n=2, θ_0=0°	K_θ=0 kcal/mol,<br>n=2, θ_0=0°
28.	CL-C2-C1-OZ<br>Cl-CT-C-O → ...		K_θ=0 kcal/mol,<br>n=2, θ_0=0°	K_θ=0 kcal/mol,<br>n=2, θ_0=0°
29.	C2...CL<br>1...2	$U_{nb}(r_{ij}) = \frac{1}{scnb} U_{vdW}(r_{ij}) + \frac{1}{scee} U_{el}(r_{ij})$	1/scee=0<br>1/scnb=0	1/scee=1<br>1/scnb=1
30.	C1...CL<br>1...3		1/scee=0<br>1/scnb=0	1/scee=1<br>1/scnb=1
31.	H1...CL<br>1...3		1/scee=0<br>1/scnb=0	1/scee=1<br>1/scnb=1
32.	H2...CL<br>1...3		1/scee=0<br>1/scnb=0	1/scee=1<br>1/scnb=1

33.	NZ...CL<br>1...4		1/scee=5/6	1/scee=1
			1/scnb=1/2	1/scnb=1
34.	OZ...CL<br>1...4		1/scee=5/6	1/scee=1
			1/scnb=1/2	1/scnb=1
35.	C2...SG<br>1...2		1/scee=1	1/scee=0
			1/scnb=1	1/scnb=0
36.	CL...SG<br>1...3		1/scee=1	1/scee=0
			1/scnb=1	1/scnb=0
37.	H1...SG<br>1...3		1/scee=1	1/scee=0
			1/scnb=1	1/scnb=0
38.	H2...SG<br>1...3		1/scee=1	1/scee=0
			1/scnb=1	1/scnb=0
39.	NZ...SG<br>1...4		1/scee=1	1/scee=5/6
			1/scnb=1	1/scnb=1/2
40.	OZ...SG<br>1...4		1/scee=1	1/scee=5/6
			1/scnb=1	1/scnb=1/2
41.	CB...C2<br>1...3		1/scee=1	1/scee=0
			1/scnb=1	1/scnb=0
42.	HB2...C2<br>1...4		1/scee=1	1/scee=5/6
			1/scnb=1	1/scnb=1/2
43.	HB3...C2<br>1...4		1/scee=1	1/scee=5/6
			1/scnb=1	1/scnb=1/2
44.	CA...C2<br>1...4		1/scee=1	1/scee=5/6
			1/scnb=1	1/scnb=1/2
45.	CB...H1		1/scee=1	1/scee=5/6

	1…4		1/scnb=1	1/scnb=1/2
46.	CB...H2<br>1…4		1/scee=1	1/scee=5/6
			1/scnb=1	1/scnb=1/2
47.	CB...C1<br>1…4		1/scee=1	1/scee=5/6
			1/scnb=1	1/scnb=1/2

Table S1 notes. This table uses the following notations for chloroacetyl atoms: C2=C^{θ}, C1=C^{η}. For complete summary of atomic names in this table see Fig. S11. Type CT for atom CB in residue CYM is equivalent to type 2C for the same atom in residue CYZ. During the reaction period T_{rxn}, all parameters indicated in the two rightmost columns are interpolated in a linear fashion, e.g. $(1/scnb)$ and $(1/scee)$ are linearly incremented/decremented with time. Parameters in the shaded cells are, strictly speaking, undefined. However, the listed values should be used for these parameters during the interpolation scheme.

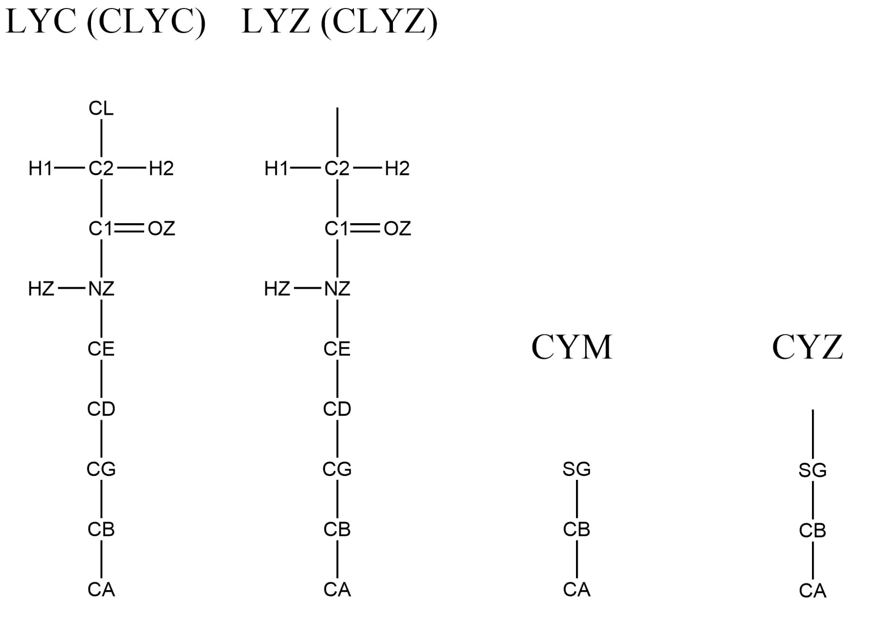

Figure S11. Key to the atom names used in Tab. S1.

Video S1. Animation representing the time interval from 230 to 760 ns in the trajectory #4 (out of eleven) showing covalent conjugation of Sos1-X' to Grb2 N-SH3. The period T_{rxn}, when the chemical reaction occurs, is shown in “slow motion” mode (during this period the timer in the screen turns red).
